# Addressing the need for individual-level exposure monitoring for firefighters using silicone samplers

**DOI:** 10.1038/s41370-024-00700-y

**Published:** 2024-07-20

**Authors:** Emily M. Bonner, Carolyn M. Poutasse, Christopher K. Haddock, Walker S. C. Poston, Sara A. Jahnke, Lane G. Tidwell, Kim A. Anderson

**Affiliations:** 1 Department of Environmental and Molecular Toxicology, Oregon State University, Corvallis, OR, USA; 2 California Air Resources Board*, Sacramento, CA, USA; 3 Center for Fire, Rescue, and EMS Health Research, National Development and Research Institutes, Inc. (NDRI)-USA, Leawood, KS, USA

## Abstract

**BACKGROUND:**

Firefighters are occupationally exposed to hazardous chemical mixtures. Silicone passive sampling devices capture unique exposures over time with minimal impact to the participant and allow for the analysis of a broad chemical space.

**OBJECTIVE:**

Silicone dog tags were worn by firefighters while on- and off-duty to measure individual exposures, identify potential occupational exposures, and assess their relation to occupational variables including fire response frequency, rank, and years as a firefighter.

**METHODS:**

Fifty-six firefighters were recruited from two fire departments with relatively high and low call volumes in the Kansas City metropolitan area to wear two different silicone dog tags as passive samplers while on- and off-duty. Each dog tag was worn for a cumulative 30-day exposure period. Extracts of the dog tags were analyzed with gas chromatography, mass spectrometry methods for 43 flame retardants (FRs), 21 volatile organic compounds (VOCs), 42 polychlorinated biphenyls (PCBs), and 63 polycyclic aromatic hydrocarbons (PAHs).

**RESULTS:**

Ninety-two total chemicals were detected, with eight chemicals not previously reported in firefighter exposure studies. Based on the magnitude and frequency of increased exposure in on-duty dog tags, relative to paired off-duty dog tags, five PBDEs and sec-butylbenzene were identified as potential occupational exposures; sec-butylbenzene and PBDE 49 have not previously been reported in firefighter exposure studies to the authors’ knowledge. Multivariate analyses for these six compounds indicated that firefighter rank, fire response rates, and years in the fire service were poor indicators of increased occupational exposure. The greatest on-duty exposures to PBDEs were found in the low-call volume department among operational firefighters. Dog tags from firefighters at the high-call volume department accounted for 75% of PCB detections; one particular fire response may have contributed to this. Additionally, there was measurable similarity in total chemical exposure profiles between paired on- and off-duty tags for some firefighters.

**IMPACT:**

- This study used personal silicone passive samplers in the configuration of dog tags worn around the neck to quantify firefighter occupational exposure in on-duty samples relative to paired off-duty samples for several chemical categories: flame retardants, VOCs, and PCBs. Five PBDEs and sec-butylbenzene were identified as potential occupational exposures, however their prevalence in on-duty tags was not associated with frequency of fire responses, firefighter rank, or years the firefighter has been in the fire service. Additionally, similarity between chemical exposures in on- and off-duty tags from the same firefighter invites further investigation into individual behaviors influencing occupational and para-occupational exposures.

## Graphical Abstract

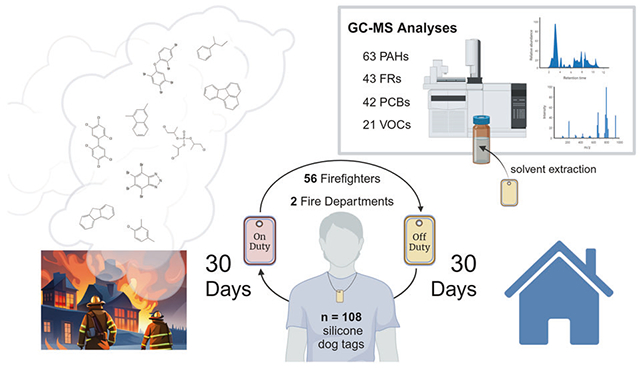

## INTRODUCTION

Firefighters are exposed to many hazards when responding to a fire, including intense heat and complex mixtures of chemicals. Despite structural firefighters wearing personal protective equipment (PPE) to mitigate these hazards, organic chemicals in smoke infiltrate PPE and can be absorbed dermally, or inhaled when the self-contained breathing apparatus is removed [1–3]. Even after a fire is extinguished, firefighters are exposed to chemicals through multiple pathways, including off-gassing from the fireground and cross-contaminated gear, truck cabs, and fire stations [2, 4, 5]. Furthermore, exposure to organic chemicals generated during a combustion event, including polycyclic aromatic hydrocarbons (PAHs) [6–8], flame retardants (FRs) [9], various volatile organic chemicals (VOCs) [10–12], and polychlorinated biphenyls (PCBs) [13] could contribute to increased risk of cancer [6, 14–17], cardiovascular disease [18], and chronic respiratory diseases [19] for firefighters. Notably, the International Agency for Research on Cancer recently promoted firefighter occupational exposures from “possibly carcinogenic” (Group 2B) to “carcinogenic to humans” (Group 1) (*IARC Monographs*, June, 2022, Lyon, France) [20]. Specifically, International Agency for Research on Cancer reports sufficient evidence of firefighter exposures causing mesothelioma and bladder cancer, and limited evidence for causing colon cancer, prostate cancer, testicular cancer, melanoma, and non-Hodgkin lymphoma [20].

Addressing firefighter health disparities requires research into possible interventions, characterization of chemical hazards, and identification of critical exposure factors, all of which demand the development of more reliable and easy-to-use chemical exposure monitoring technologies for on-duty firefighters. Silicone passive sampling is a promising technology to serve this purpose. Since its first application in 2014, personal silicone passive sampling has been successfully applied in comparing personal chemical exposures in diverse scenarios, primarily as silicone wristbands [21]. Previous studies utilizing silicone passive samplers on the fireground have measured exposure to PAHs [22–28], as well as other volatile and semi-volatile organic chemicals including (but not limited to) phthalates, pesticides, flame retardants [22–24, 29], and in one pilot study, PFAS [24]. Silicone passive samplers are specifically advantageous sampling tools in disaster scenarios and emergency response due to their ease of collection for participants [30]. Unlike an active air sampler, silicone samplers are light and do not require pumps or batteries to function; they are also transportable at ambient temperatures. Additionally, the silicone polymer passively captures time-weighted concentrations, allowing scientists to capture unpredictable exposure events, such as fires [31]. In contrast, a biological sample (e.g., blood, urine) can be challenging to time appropriately for capturing exposure during an emergency response and are generally invasive, although it should be acknowledged that biological samples are important measures of internal dose [1].

The sample set analyzed in this study was the first to use silicone passive samplers configured as a military-style dog tag, as reported by Poutasse et al. in 2020 and 2022 [22, 29]. The silicone dog tag is worn around the neck, which is a particularly vulnerable location for firefighter dermal exposure in terms of PPE penetration by chemicals, and the relative thinness of the skin at this location which allows for greater rates of dermal absorption [23, 32]. Analyses in this study include quantitation of silicone dog tags for FRs, VOCs, and PCBs with some comparative analysis to PAH data reported by Poutasse et al. [22, 29]. The investigated chemical categories and specific analytes, reported in Table 1, were informed by suspected toxicological relevance, chemical categories of importance in previous firefighting exposure studies, and available chemical standards. Analytes were also selected with the intention to span a large range of physical-chemical properties (predicted LogK_oa_ range: 3.8–11.7; molecular weight range: 104.2–722.5 g/mol). In the context of other silicone passive sampling studies investigating firefighter exposures, which have not quantified PCBs and infrequently quantify VOCs, this combination of analyses expands the field’s understanding of the capacity of these samplers to quantify diverse chemical mixtures [21].

FRs are a class of chemical defined by their use. Poly (penta- and octa-) brominated diphenyl ethers (PBDEs) were common constituents of FR mixtures in the 20th century, but were phased out of use in commercial products in the US (as of 2004) due bioaccumulation and persistence concerns, plus links with liver, thyroid, and neurodevelopmental toxicity [33]. However, they persist in products such as upholstered furniture, building materials, and electronics. Currently, organophosphate FRs and alternative brominated FRs are added to manufactured goods, which have also been associated with health risks including reproductive toxicity and lesion formation in the brain and kidneys [33]. Elevated levels of FRs have been measured in the air during a fire and in fire stations, as well as biomarkers of FRs in serum and urine of firefighters [9, 34–38].

VOCs, defined by their high vapor pressure and low water solubility, are generated during combustion, and are used in a variety of manufactured products (e.g., paints, adhesives, building materials) [39]. VOCs have been measured under PPE during a fire, off-gassing from gear following a fire, and elevated VOCs (and biomarkers) have also been measured in firefighter breath samples and serum following a fire [5, 10, 32]. Various VOCs are carcinogenic, irritants, or related to other toxic endpoints (e.g., respiratory illness, nervous system damage) following acute or chronic exposures [39, 40].

PCBs persist in electrical equipment (e.g., capacitors, transformers), as well as building materials in the United States following their use between about 1930 and 1977 [41, 42]. Potential for firefighter exposure to PCBs has been demonstrated with measures of elevated levels of PCBs in firefighter serum [13, 43]. PCBs and their metabolites are recognized as mutagenic and Group 1 carcinogens, and are associated with numerous other adverse health effects [44].

Quantifying FRs, VOCs, and PCBs in the silicone dog tags worn by on-duty firefighters will provide relevant exposure data and continue to test the limits of this relatively new technology in measuring classes of chemicals with different properties, fate, and exposure routes. The aim of this study is to expand the chemical space considered for firefighter occupational exposure, evaluate models of measured occupational exposures in response to common exposure surrogates, and discuss the merits and challenges of using personal silicone passive samplers to measure firefighter chemical exposures. It is hypothesized that on-duty exposure exceeds off-duty exposure for multiple chemicals not previously quantified in firefighter exposure studies and that firefighter rank, years as a firefighter, and frequency of responses to fires are indicative of occupational exposure. Additionally, dissimilarity within and between exposure profiles of individuals was compared to explore variability in total exposure between on- and off-duty samples from the same firefighter and inform individual variability in exposure.

## MATERIALS AND METHODS

### Materials

All laboratory glassware was washed in an automatic dishwasher with detergent and rinsed with 18MΩ·cm ultrapure water, then dried in an oven at >300 °C for 12 h. Optima-grade solvents from Fisher Scientific (Pittsburg, PA, USA) were purchased. Analytical standards were sourced from Accustandard (New Haven, CT, USA), Chiron (Trondheim, Norway), TCI America (Tokyo, Japan), SantaCruz Biotechnology (Dallas, TX, USA), and Sigma-Aldrich (St. Louis, MO, USA). Polytetrafluoroethylene bags and closures used for transporting samples in an air-tight environment were purchased from Welch Fluorocarbon, Inc. (Dover, NH, USA) [45].

### Silicone dog tag preparation and extraction

Silicone dog tags were purchased from https://24hourwristbands.com (Houston, TX, USA) and prepared with previously documented procedures [22, 46]. The preparation involved vacuum oven conditioning at 300 °C for 12 h at 0.1 Torr (oven model: Blue-M POM18VC; pump model: Welch Duoseal, no. 1405). After preparation, quality control samples were reserved, and dog tags were stored in sealed metal containers at 4 °C. For transportation, dog tags were packaged in polytetrafluoroethylene bags.

Following deployment, the silicone dog tags were rinsed with 18MΩ ·cm ultrapure water and isopropanol to remove the particulate-bound chemical fraction on the exterior before storing dog tags in amber glass jars at −20 °C, as previously reported [46, 47]. Half of each dog tag remained in storage, while the other half was solvent extracted with ethyl acetate and further processed through solid phase extraction [22, 46–48]. Refer to the SM for detailed procedure and chemical standard recoveries.

### Firefighter participants and protocol

Informed consent was obtained from all participants, and protocols were approved by the National Development and Research Institutes, Inc. (NDRI) Institutional Review Board (IRB00000634; Oregon State University IRB Deferral 8313). As reported by Poutasse et al., 57 firefighters were recruited from two fire departments in the Kansas City metropolitan area [22]. One department historically received more fire calls than the other and will be referred to as the “high call volume” (>12 fire calls per month on average; 30 firefighters) and the “low call volume” (<2 fire calls per month on average; 27 firefighters) departments. To avoid capturing chemicals in firefighter PPE from previous exposures, firefighter participants were provided with new PPE, including turnouts and hoods (GXTREME 3.0, Globe Manufacturing Company, LLC, Pittsfield, NH, USA; Quest Particle Barrier Hoods, Quest Fire Apparel Inc., Saratoga Springs, NY, USA) [22].

Firefighter participants completed a questionnaire with demographic, occupational, and exposure information at the beginning of the study. During sample collection (November 2018 to April 2019), all “fire attacks,” or fires that an individual firefighter responded to, were recorded. Firefighters wore one silicone dog tag on an elastic necklace during 30 on-shift days, and a separate tag during 30 off-shift days. Dog tags were stored in their unique polytetrafluoroethylene bag whenever they were not in use. Participants were asked to wear the dog tags at all times, including while sleeping and showering. When donning PPE, firefighters were asked to keep the dog tag over their clothes, but under their turnout gear. Participant compliance rates and demographic information are summarized in Poutasse et al. [22].

### Instrument analysis

Three different instrument methods are used to analyze sample extracts for FRs, VOCs, and PCBs, all of which relied on gas chromatography and mass spectrometry using selective ion monitoring mode with electron impact ionization. Instrument concentrations are quantitatively corrected for laboratory processing losses using recoveries of extraction surrogates. Analytes can be found in Table 1, and limits of detection and quantitation, internal standards, extraction surrogates, and method parameters are detailed per method in Tables S1–S7. Methodology for quantifying and reporting PAH data is detailed in Poutasse et al. [22].

### Quality control

Quality control (QC) samples comprised 32% of all analyzed samples. QC dog tags were collected at different stages of sample preparation, deployment, lab processing, and during instrumental analyses [22]. One FR, triphenyl phosphate, was detected in one QC sample. This QC sample was associated with a single participant sample, which was corrected with background subtraction. Background corrections for VOCs in samples were performed using concentrations found in the corresponding laboratory processing blank (*n* = 2), which are exposed to all laboratory processes (see SM). Hexadecane was detected at large and variable concentrations in all QC samples and will therefore be omitted from data analysis. No PCBs were detected in any QC samples.

### Data analysis

All statistical analysis and data visualization is performed in R version 4.3.1. The “vegan” package in R was used to create distance matrices, perform PERMANOVA (using the adonis2 function, 999 permutations), and create nonmetric multidimensional scaling (NMDS) plots [49]. Note that PAHs are included in some figures for context (reported in [22]), but the focus will be on data from the other three chemical categories, which have not been previously discussed in the literature.

#### Potential occupational exposures.

To address the first hypothesis, a combination of sign tests and magnitude of positive differences between paired samples were used to identify “potential occupational exposures.” Two-sided sign tests were performed for all chemicals detected in at least six individuals’ dog-tags to test the alternative hypothesis that the median difference between paired on- and off-duty dog-tags is non-zero. In cases where one of the two paired dog tags was below the limit of detection, the difference between the limit of detection and the detected concentration in the other tag was calculated. In cases where both paired samples were below the limit of detection, the difference was characterized as zero. The magnitude of “occupational exposure” was described using Log_2_(fold-change (FC)) between paired on and off-duty tags. Criteria for chemicals to be defined as “potential occupational exposures” include: six or more individuals with detections, a p-value less than 0.05 for the sign test, and a mean Log_2_(FC) greater than 0.5.

To determine if a chemical was quantified in previous firefighter exposure studies, a literature review of all detected chemicals was performed through Google Scholar using search strings constituting of “firefighter” and all reasonable chemical synonyms, including the chemical CAS number. Chemical synonyms were determined with the Sciome SWIFT-review tool and are documented in Table 2 [50]. The numbers of search results were documented, and the resulting publications were manually reviewed.

#### Assessing exposure surrogates.

A PERMANOVA model was constructed to assess the relationships between on-duty exposure and the firefighters’ rank, years in the fire service, and number of fire attacks they participated in while wearing their sample, stratified by department. A Euclidean distance matrix was used, derived from a matrix of Log_2_(FC) between a firefighters’ on- and off-duty tag for the six chemicals deemed “potential occupational exposures” (see “Potential occupational exposures”). Homogenous dispersion among groups was confirmed. NMDS analysis was used to visualize the distance matrix.

#### Individual variability.

To explore inter- and intra-individual exposure variability, Bray Curtis dissimilarity scores were computed for pairwise comparisons of all samples, and intra-individual scores (on- vs. off-duty dog tag from the same firefighter) were compared to the distributions of inter-individual scores. NMDS analysis was used to visualize the dissimilarity matrix. To account for left-censored data from different instrumental methods, chemical concentration data was converted into ordinal quartiles: very low, low, medium, and high exposure. If the percentage of observations below the LOD was less than or equal to 25%, empirical percentiles of 25, 50, and 75% were used as thresholds for the quartiles. If the percentage of observations below the LOD was greater than 25%, all observations below LOD were assigned to the very low category and the remaining observations were divided equally into low, medium, and high exposure. All thresholds are reported in Table S12.

## RESULTS

### Compliance data

Compliance and demographic data have been previously reported in Poutasse et al. [22]. Briefly, all firefighters returned on-duty tags (*n*_on-duty_ = 57; 100% compliance), and all but three firefighters returned their off-duty tags (*n*_off-duty_ = 54; 95% compliance).

### Chemical exposure profiles

**Fig. 1:**
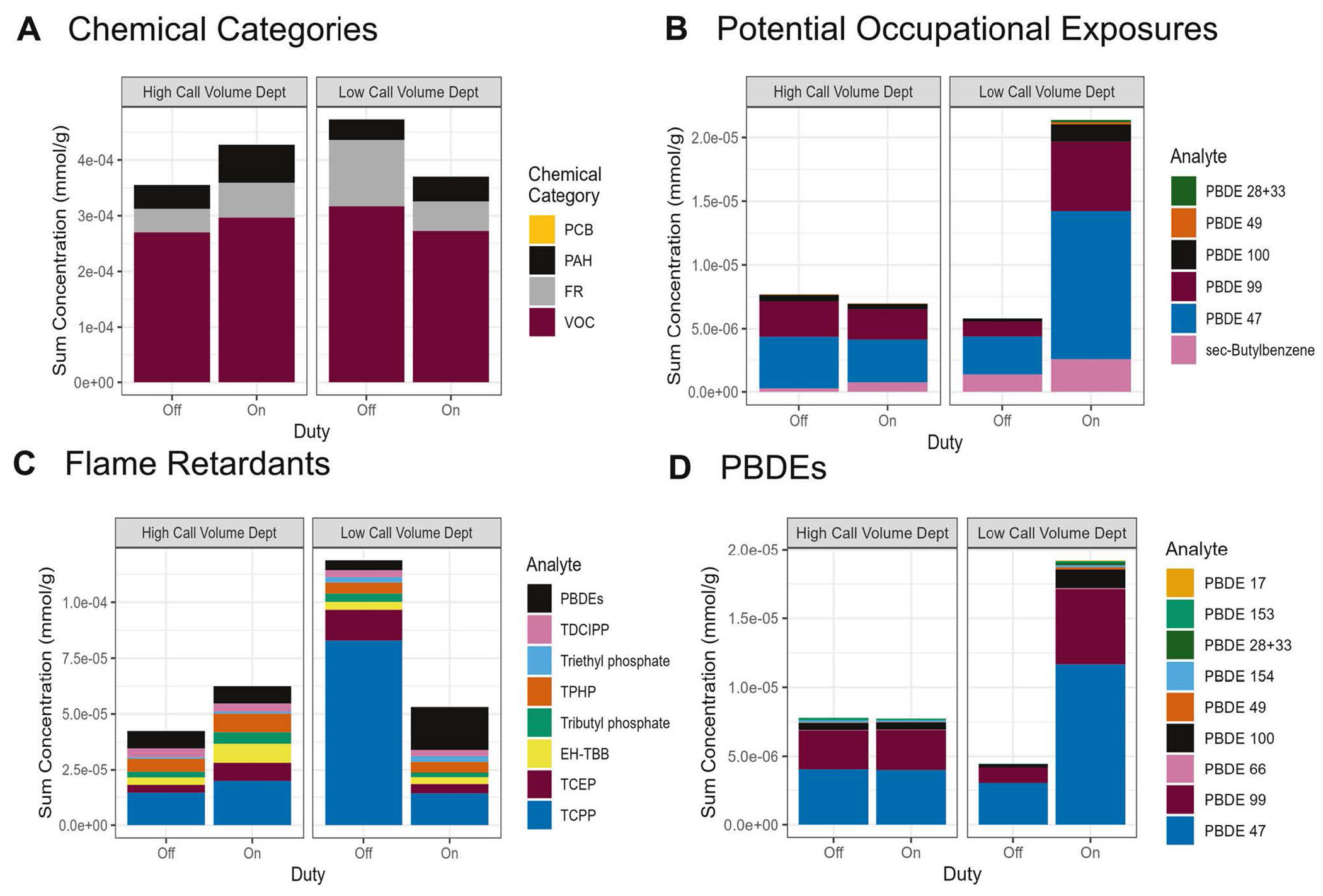
Sum chemical concentrations by department and duty status. **A** Sum concentration of chemical categories: VOCs, FRs, PAHs, PCBs, and VOCs in mmol/g silicone. **B** Sum concentrations of potential occupational exposures in mmol/g silicone. Sum concentrations of FRs by department and duty status for (**C**) all FRs and (**D**) just PBDEs in mmol/g silicone.

**Fig. 2:**
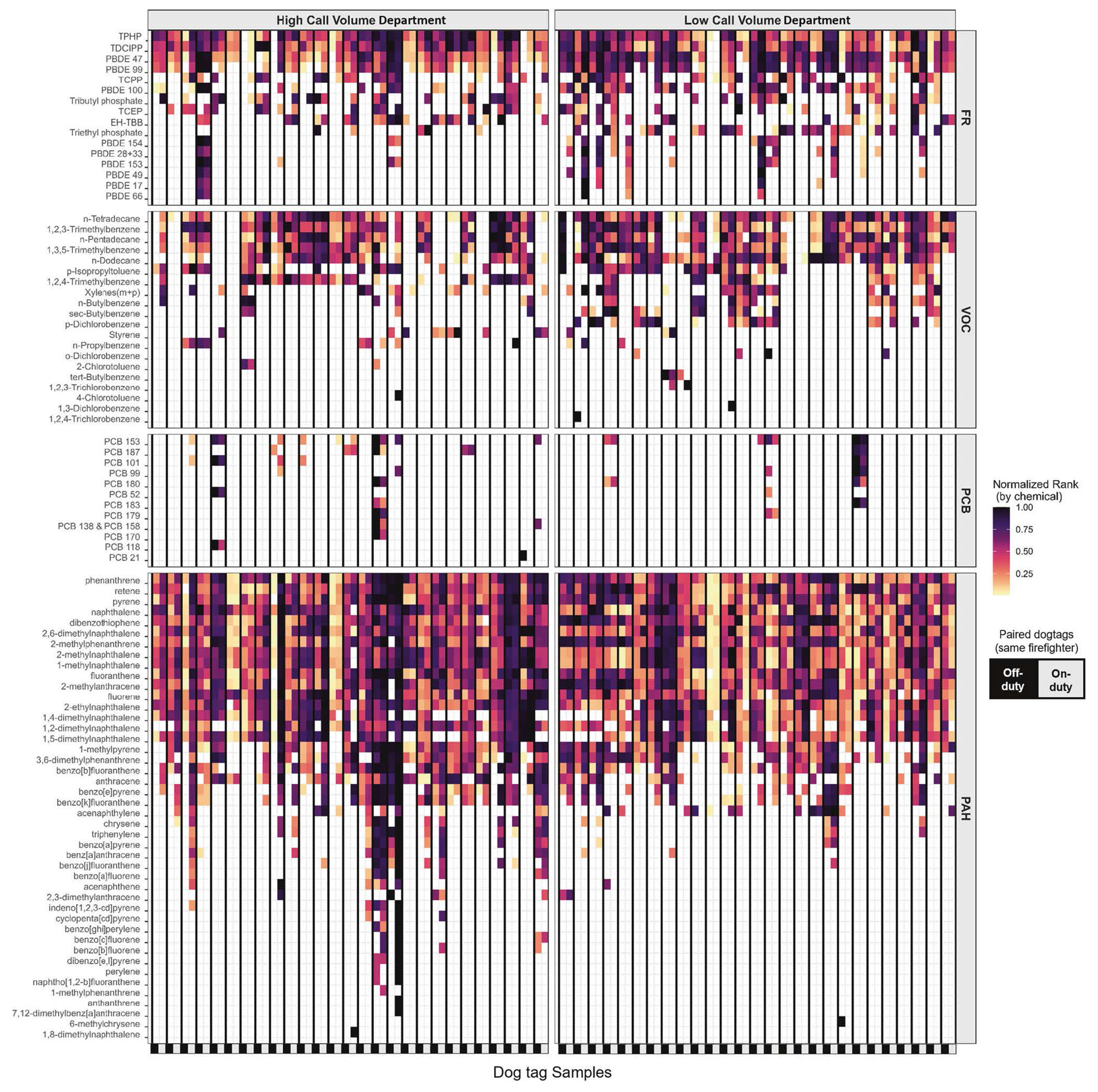
Personal chemical exposure profiles. Normalized concentration rank (0–1) for each detected chemical is represented in the heatmap, with the darkest squares corresponding to the highest relative rank of the sample for the specified chemical; white space represents chemicals below the limit of detection. Chemicals constitute rows, and individual samples (*n* = 108) are the columns, with paired off- (left, black) and on-duty (right, gray) tags grouped within the bolded vertical lines. The heat map is grouped by fire department (columns) and chemical category (rows).

Ninety-two chemicals were detected in at least one sample, and 59 chemicals were detected in the dog tags of at least six firefighters; to the authors’ knowledge, the following chemicals have not previously been reported in firefighter exposure studies: PBDE 17, PBDE 49, 2-ethylnaphthalene, 2-methylanthracene, 1,2,3-trimethylbenzene, 1,3,5-trimethylbenzene, n-butylbenzene, and sec-butylbenzene. The summed molar mass of VOCs captured was the largest, despite having the fewest analytes, followed by FRs, PAHs, and lastly PCBs. The sum concentrations of chemical categories under different sampling conditions are visualized in Fig. 1A. Individual chemical profile patterns can be observed in Fig. 2, which shows detected chemicals across all four chemical categories. Summary statistics for chemicals detected in at least six samples are given in Table 2.

#### FRs.

Sixteen FRs were detected in at least one sample, including six organophosphate ester flame retardants, nine PBDE congeners, and 2-ethylhexyl-2,3,4,5 tetrabromobenzoate (EH-TBB). Triphenyl phosphate (TPHP) and tris(1,3-dichloro-2-propyl)phosphate (TDCIPP) are the most frequently detected FRs (both found in 102 of 108 dog tags), while tris(2-chloroisopropyl)phosphate (TCPP) and tris(2-carboxyethyl)phosphine (TCEP) contribute the largest total molar mass, especially for off-duty low call volume department firefighters as seen in Fig. 1C. The off-duty sum molar mass for TCPP is largely attributable to four individuals constituting 79% of the mass, with one sample at 3.75 × 10^−5^ mmol/g silicone (57%). The PBDEs contributing the greatest sum mass belong to the penta-BDE mixture, including PBDEs 99, 47, and 100 (Fig. 1D).

Thirteen, nominally half, of low call volume department firefighters were exposed to a mixture of PBDE congeners unique to their on-duty dog tag (Fig. 2). Among these, PBDE congeners 17, 28 + 33, 49, 66, 153 and 154 were detected much less frequently at the high call volume department, barring an individual firefighter with relatively high exposure in both their on- and off-duty samples.

#### VOCs.

Twenty-one VOCs were detected in at least one sample. By molar mass, VOCs were approximately ten times more prevalent than any other chemical category (Fig. 1A) at both departments in on- and off-duty tags. Straight-chain alkanes were the most frequently detected category of VOC, with the greatest proportion of mass overall, followed by trimethylbenzenes (Fig. S1).

At the high-call volume department, the number of detections in on-duty tags was greater than in off-duty tags for 11 of the 13 VOCs detected in the tags of at least six different firefighters. This trend is not evident at the low call volume department. *para*-dichlorobenzene was detected in 11 on-duty tags at the low call volume department, but only in one sampler at the high call volume department (Table 2). Differences in concentrations of VOCs between on-and off-duty tags are highly variable among individuals; some firefighters have greater sum VOC exposures and detection frequencies while on-duty, but others have greater exposure while off-duty (Fig. S2).

#### PCBs.

The total concentrations of detected PCBs are an order of magnitude smaller than the other chemical categories, making them almost imperceivable in the proportion plot in Fig. 1A; proportions of molar mass for individual congeners are documented in the SM (Fig. S1). Twelve PCB congeners were detected, including a co-eluting congener (PCB 138 or 158). PCB 153 is the most frequently detected congener, present in 17% of all dog tag samples. Only one of the detected PCBs is classified as “dioxin-like” or coplanar, (PCB 118) and was detected in the two samples worn by the same firefighter.

The frequency of PCB detections is also low; out of 57 firefighters, only 16 (28%) had at least one PCB detection in either of their dog tags, 12 of which are from the high call volume department. Roughly 50% of firefighters with PCB detections had quantifiable concentrations of PCB congeners in both on and off-duty dog tags. In many of these cases, the congeners observed in the paired dog tags were overlapping (see PCB panel of Fig. 2).

Four firefighters had only on-duty detections, and three firefighters had only off-duty detections. For those firefighters with detections in both paired tags, no consistent differences were found in summed PCB concentrations between paired on- and off-duty samples. However, the four firefighters with the greatest number of detections and largest sum PCB concentrations had higher exposures in their off-duty tags.

### Potential occupational exposures

The proportion of positive differences between on- and off-duty dog tags, sign test results, differences in concentration between on- and off-duty dog tags, and literature review results, are summarized in Table 2 for all chemicals detected in samples from at least six different firefighters.

**Fig. 3:**
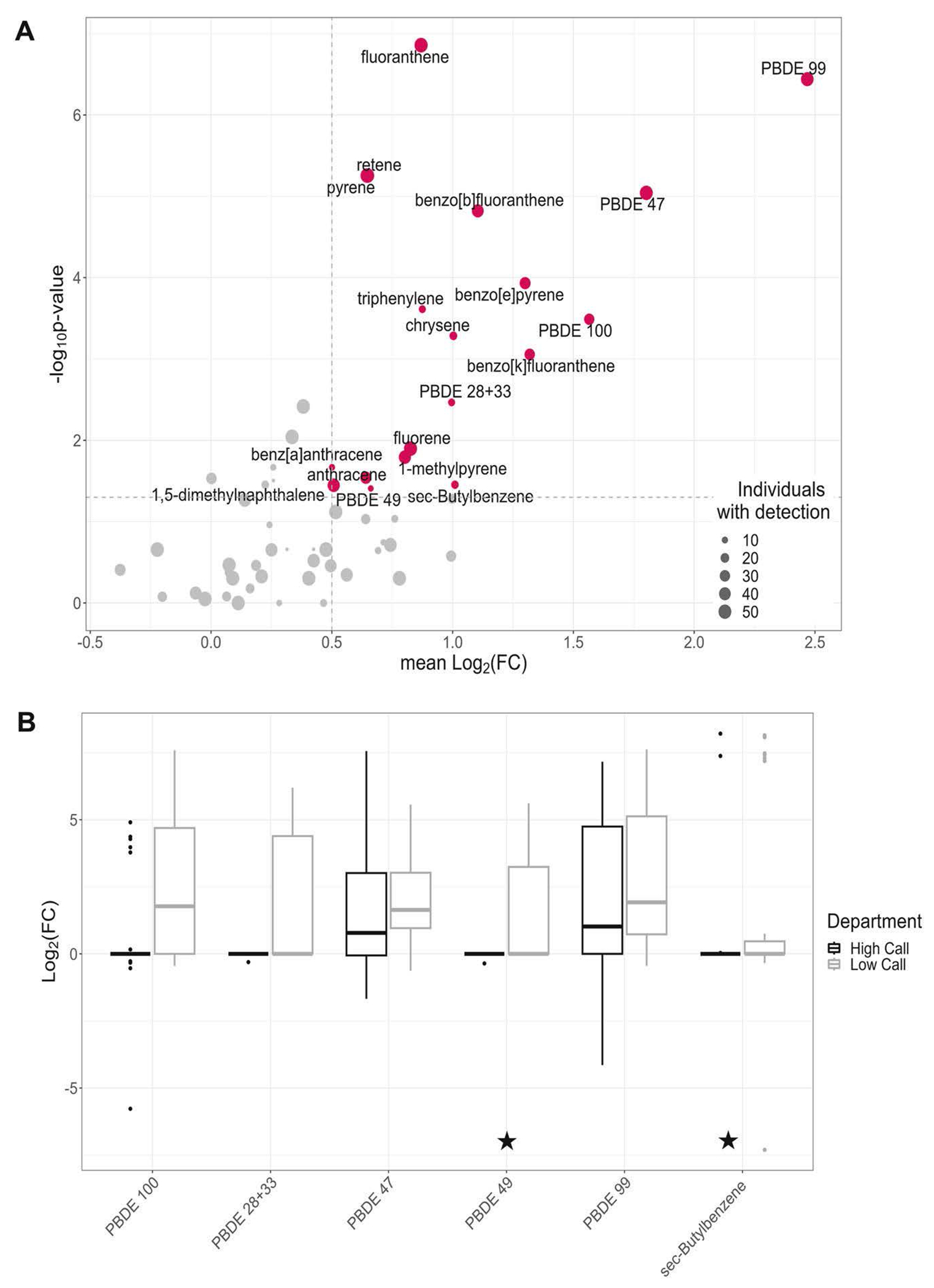
Potential occupational exposures. **A** Red points highlight chemicals identified as potential occupational exposure based on thresholds for mean Log_2_(FC) (>0.5) and −Log_10_(p-value) (*p* value < 0.05 from a one-sided sign test). Size of point represents number of pairs with at least one concentration above the limit of detection. **B** Distribution of Log_2_(FC) for chemicals (excluding PAHs) identified as potential occupational exposures in (**A**), stratified by fire department (see Table 2 for *n*). Starred chemicals have not previously been characterized in a firefighter exposure study (PBDE 49 and sec-butylbenzene).

Chemical exposure was generally greater in on-duty tags than off-duty tags;only three out of the sixty chemicals found in at least six firefighters’ tags (TDCIPP, TCPP and naphthalene) had proportions less than 50% for positive differences between paired on- and off-duty tags, none of which were significant with the sign test. Based on pre-defined criteria, five PBDE congeners (28 + 33, 47, 49, 99, and 100) and sec-butylbenzene, a VOC, were identified as potential occupational exposures (Fig. 3). Of the chemicals identified as potential occupational exposures, sec-butylbenzene and PDBE 49 have not previously been quantified in firefighter exposure studies to the authors’ knowledge. Proportions of sum concentrations for the six potential occupational exposures are shown in Fig. 1B.

The boxplots in Fig. 3B show the variability in Log_2_(FC) distribution shapes between chemicals and fire departments. PBDEs 47 and 99 have the strongest evidence in support of them being occupational exposures based on magnitude of difference between paired tags (3.5 times greater and 5.7 times greater concentration on average), proportion of positive differences between paired tags (81% and 86%), and number of firefighters with detections (52 and 49 individuals). Sign-test *p* values for PBDEs 47 and 99 were 9.1 × 10^−6^ and 3.6 × 10^−7^ respectively.

### Assessing exposure surrogates

Relevant exposure surrogate measures from the firefighters are summarized, along with detection frequencies and Log_2_(FC) distributions, for the six identified potential occupational exposures in Tables S10–S11. Additionally, univariate comparisons of Log_2_(FC) distributions are shown for each of the questionnaire variables of interest across the six potential occupational exposures (Fig. S3). Median Log_2_(FC) of PBDEs 28 + 33 and 47 appeared to increase with greater numbers of fire attacks and decrease with more years in the fire service based on binned responses, but only for firefighters at the low call volume department (Fig. S3).

In the multivariate PERMANOVA analysis, all explanatory variables had small effect sizes and p-values exceeding 0.05 in the PERMANOVA model. Explanatory variable levels also did not cluster well in nonmetric multidimensional scaling plots (Fig. S5), suggesting that these metrics did not serve as effective surrogates of the potential occupational exposures.

Beyond the identified potential occupational exposures, PCBs were detected in the on-duty tags of all five operational firefighters that responded to a particular house fire on 1/25/2019 from the high call volume department. Two other firefighters of different ranks responded to the fire, although likely in a different capacity, and didn’t have any PCB detections.

### Individual variability

Some of the patterns seen for individual firefighters have been mentioned in the chemical class results and can be observed in Fig. 2. For example, there are cases with FRs, VOCs, and PCBs, where an individual has unique detections for a chemical in both their on- and off-duty sample. This is especially evident for the PCBs, which have infrequent detections, but there is often overlap in congeners detected in on- and off-duty tags.

**Fig. 4:**
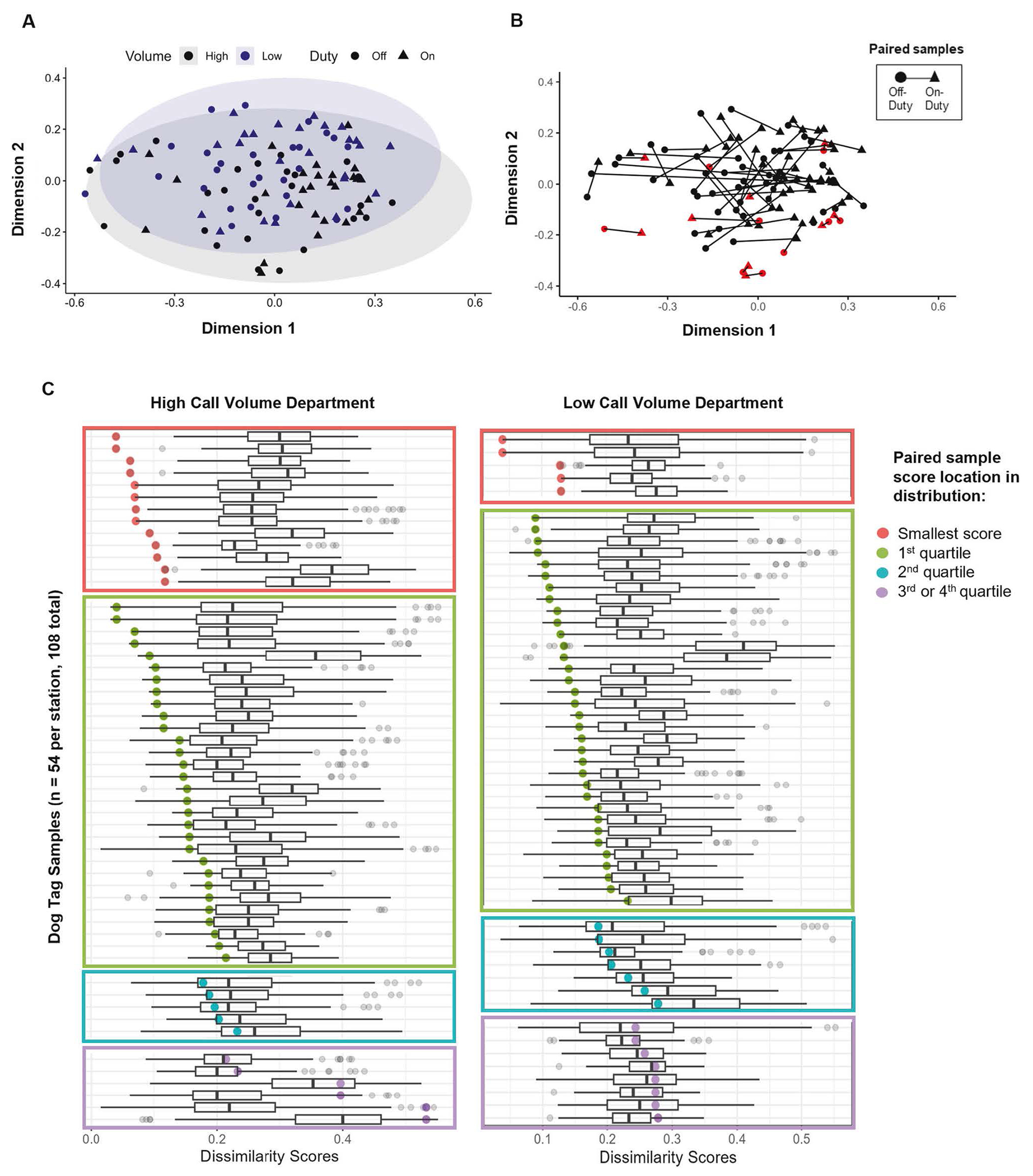
Bray Curtis dissimilarity scores for individual samples and chemicals detected in at least 50% of samples. **A** and **B** are the same NMDS plot (stress = 0.2) with different annotations. Circle points indicate off-duty samples and triangles indicate on-duty samples. **A** Department (high call volume = black; low call volume = blue) corresponding to samples is shown, with ellipses illustrating the concentration of points from that department. **B** Paired on- and off-duty samples are connected with a line and red points highlighting 18 samples most similar to their pair. **C** Each row within each department represents a unique sample, with a boxplot representing the distribution of dissimilarity scores from pairwise comparisons to all other samples. The paired, or intra-individual sample dissimilarity score is highlighted as a colored point overlayed on the boxplot.

To further investigate the similarities and differences between paired and non-paired samples, Bray Curtis dissimilarity was computed for quartiles of chemicals detected in 50% of samples. The NMDS plots in Fig. 4A illustrate minimal clustering of samples by department or duty status, but B a frequent grouping of paired on- and off-duty samples from the same individual. It should be noted that there are also cases where the lines connecting paired samples are quite long and overlapping, suggesting greater dissimilarity between those samples. This information is detailed in Fig. 4C.

Across all samples (*n* = 108), 18 (17%) had the smallest Bray Curtis dissimilarity scores with their paired sample, from the same firefighter, meaning they are the most similar (red points in Fig. 4B, C). Another 64 (59%) samples had paired dissimilarity scores smaller than 75% of all other sample scores (green points in Fig. 4C). Only 14 samples (13%) had paired dissimilarity scores that exceeded the median score (purple points in Fig. 4C). This analysis indicates that paired samples were commonly more similar in chemical composition and magnitude of chemical concentrations to each other than to other samples, even within the same duty-status and fire department.

## DISCUSSION

### Potential occupational exposures for structural firefighters

Since this study’s original investigation of PAH exposure and semi-quantitative screening for over 1500 organic chemicals, the quantitative analysis was expanded to a broader scope of chemicals, including FRs, VOCs, and PCBs [22, 29]. PBDE 17, PBDE 49, 2-ethylnaphthalene, 2-methylanthracene, 1,2,3-trimethylbenzene, 1,3,5-trimethylbenzene, n-butylbenzene, and sec-butylbenzene were detected in this study, but have not previously been reported in firefighter exposure studies to the authors’ knowledge and should be considered in future monitoring studies.

Across all four chemical categories investigated, there were 19 chemicals identified as potential occupational exposures based on criteria that included proportion of positive differences and magnitude of fold-change between on- and off-duty paired samples. More than half of these chemicals were PAHs, and were previously discussed by Poutasse et al. [22]. As common combustion byproducts, PAH mixtures are commonly detected on the fireground, although the number of analytes investigated through this study is beyond the typical scope. Recently, Baum et al. have provided additional support for the detection of many PAHs in these silicone dog tags, including alkylated PAHs [25]. However, this is the only study to date that has detected 2-ethylnaphthalene among firefighter exposures.

Beyond PAHs, five FRs (all PBDEs) and sec-butylbenzene, a VOC, were identified as potential occupational exposures by the criteria defined in this study. Other studies support PBDEs being occupational exposures for structural firefighters [9, 34, 36–38, 51], with studied sources including the firetruck, fireground, deployment gear, and residual contamination in the fire station. PBDEs 47 and 99 are consistently found at relatively high concentrations in these studies, which agrees with the results in Fig. 3, in which PBDEs 47 and 99 are the strongest candidates for occupational exposure. PBDEs 47 and 99 also constitute the largest mass proportion of sum PBDEs measured in this study (Fig. 1D). PentaBDE is a commercial mixture of PBDEs phased out in commercial products in the US in 2004 due bioaccumulation and persistence, as well as to links with liver toxicity, thyroid toxicity, and neurodevelopmental toxicity [33]. It was used heavily in polyurethane foam products, including upholstered furniture [52]. The most abundant components of this mixture are PBDEs 47 and 99, but also includes PBDEs 28 and 100, two of the other identified potential occupational exposures (PBDE 28 uncertain; co-elutes with 33). Correlation in concentrations of pentaBDE constituents was also observed, which provides further support that firefighters were exposed to a commercial mixture (Fig. S6).

Interestingly, another analyte with some evidence of being occupational in source, EH-TBB, is a PBDE replacement, found in commercial flame-retardant formulations in the United States, with production beginning in 1995 and on-going high-volume production as part of Firemaster^®^ 550 [53]. While novel brominated flame retardants are not as well characterized as PBDEs, Brown et al reported elevated levels of EH-TBB in fire department dust versus other indoor environments, providing some additional support towards this compound being an occupational exposure [54]. Generally, PBDEs were more prevalent in on-duty dog tags from the low call volume department, while EH-TBB was more prevalent in on-duty tags from the high call volume department.

Sec-butylbenzene, the only VOC identified as a potential occupational exposure, is poorly studied and has not previously been reported in firefighter exposure studies. It is used as a solvent for coating compositions and a plasticizer and can be a combustion byproduct. There is currently inadequate data to assess sec-butylbenzene’s carcinogenic potential according to the CompTox Dashboard (version 2.2.1; accessed 9/26/2023) [55]. Further exposure characterization on the fireground and toxicity data should be pursued in future studies.

### Assessment of exposure surrogates

There were no observable increases in occupational exposure based on rank, years as a firefighter, or number of fires responded to while wearing the on-duty dog tag based on a PERMANOVA model. This is an important result, as these factors are often used as chemical exposure surrogates for firefighters. However, the data does suggest that exposures while at work are influenced by an individuals’ environment, which depends on the fire station that they work at, the individual fires they respond to, and their rank. For example, at the high call volume department, PCBs were detected in the on-duty tags of all five operational firefighters that responded to a house fire on 1/25/2019. Two other firefighters of different ranks responded to the fire, although likely in a different capacity, and didn’t have any PCB detections.

Another example of exposure that appears to be dependent on a combination of variables, is to xylenes (m + p), which was influenced by department and rank. The most notable exposure was for on-duty chiefs at the high call volume department (median Log_2_(FC) for chiefs is 7.48; median Log_2_(FC) for all other ranks is zero). Chiefs in the fire service typically have their own offices within the station and take separate vehicles to fire responses; it is possible that one of these unique environments contained xylenes. Some potential sources of xylenes are printers, automobile exhaust, paints, or adhesives.

PBDEs made up a large proportion of the potential occupational exposures that were identified, but this was generally driven by a difference in on- and off-duty detections at the low call volume department (Fig. 3B). PBDEs detected at the low call volume department were primarily in the dog tags of operational firefighters and captains, not chiefs. There was also a trend of increased Log_2_(FC) with increased fire counts and decreased years as a firefighter for some of the PBDEs at the low-call volume department specifically (congeners 28 + 33 and 47).

It should be noted that all these variables (rank, fire counts, years) are related; someone in the fire service longer could be promoted to captain or chief. Fire chiefs take on more administrative roles leading them to inhabit different spaces and hours in the department, and in this dataset, chiefs responded to fewer fires on average. The department itself, rather than fire suppression activities, could contribute to PBDE exposure. There could be furniture, building materials, or other consumer products at the department containing PBDEs, specifically the pentaBDE mix discussed in Section 4.1. This idea is corroborated by data from Levasseur et al., in which relevant PBDE congeners were found to be higher silicone samples worn by firefighters while on-duty (up to seven times), relative to off-duty, but PBDE exposures were not elevated for firefighters that responded to fires relative to those that did not [24]. Additionally, exposure could be indirectly facilitated through the department due to inadequate segregation of gear storage in the station [35]. This could result in chronic off-gassing of compounds, as proposed by Banks et al. [38, 51].

### Individual exposures

Individual samples, and firefighters, had unique chemical exposure profiles. Figure 2 visually demonstrates that no two profiles look the same, and the plots in Fig. 4 suggest that intra-pair dissimilarity scores are often smaller than inter-pair dissimilarity scores, even within the same department and duty status. In fact, 17% of all samples were more similar to their paired sample from the same firefighter, than any other sample. Furthermore, 76% of samples were more similar to their paired sample than 75% of all other samples and only 14 (13%) of samples were less similar to their paired sample than 50% of all other samples. Combining this result with the fact that exposure surrogates (rank, years as a firefighter, or frequency of fire responses) did not perform well as a proxy for exposure in this study, it seems that measuring personal chemical exposures is informative and important. The individuality of exposure has been discussed in other studies as well, including other personal silicone passive sampling studies employing repeated measures [56]. Similarity between dog tags from the same firefighter provides important information for future study designs involving silicone passive samplers, for example, affirming the collection of samples from on-and off-duty timepoints and treatment of samples from the same firefighter under different conditions as paired, rather than independent. Further work should also be done to elucidate the reason for similarity between paired samples.

Some individuals had relatively low or high exposures across all detected chemicals, which could be in part due to more effective PPE usage or decontamination efforts after a fire. Individual exposures are not limited to fire suppression activities, but also result from the home environment, habits (e.g., smoking, cleaning, driving) and consumer product usage. As discussed, even some of the identified potential occupational exposures in this study could result from consumer products including furniture. Furthermore, the overlap in exposures between on- and off-duty tags of some individuals suggests that there could be carry-over of exposure between work and home. On the other hand, some individuals had more dissimilar on- and off-duty dogtags, which might suggest there are actions those individuals are taking which reduce their para-occupational exposures.

Current literature points to several critical interventions for reducing chemical exposure related to firefighting activities and avoiding cross-contamination:

1. Gross decontamination of PPE with soap following a fire response [9].
2. Segregate gear, and do not store in the cab of the truck when transporting back to the station; avoid transporting in private vehicles [4].
3. Following guidelines from the NFPA on laundering gear, and storage of gear in a location that is less likely to off-gas in the station [2, 5, 9, 36, 51].
4. Usage of SCBA, even for engineers and during overhaul [57].

Future studies could specifically utilize silicone passive samplers to investigate the impact that compliance with these various practices has on individual exposures. The involvement of multiple departments and geographies could also expand knowledge of individual firefighter exposures across the US and in other countries and help elucidate best practices for reducing exposure. To this end, it will be important to capture information in future studies about individual fires, PPE compliance, specific operations participated in during each fire, individual and departmental practices around decontamination and gear storage [36].

### Limitations and strengths

#### Limitations.

Each of the chemical categories investigated in this study came with unique quantification challenges and results. Most notably, VOCs have relatively fast uptake (and release) rates into the sampling material, which results in a shorter linear-uptake phase, and may not be suited to sampling occurring over more than a few days [58]. Uptake rates for individual detected VOCs were used to determine the exposure window expected to contribute to concentrations measured in the firefighters’ dog tags (calculations included in the SM). Estimated time to equilibrium between the environment and the silicone passive sampler range from 18 h (toluene) to 19 days (pentadecane), with most detected VOCs in the range of two to three days. The evaporation steps involved in the extraction processes that were used to process the samples also reduce recovery of volatile compounds from the sampling material in the final sample extract, which reduces the sensitivity of the analysis. Different analytical techniques, such as thermal desorption, could improve recoveries and sensitivities for these more volatile chemicals.

It is also important to acknowledge that the sample size was limited and that firefighters were from two fire departments in the same city; this does not necessarily represent the larger firefighter population. For the PERMANOVA models, there were some groups with smaller or uneven sample sizes, leading to reduced statistical power. For more confidence in the results, more samples within each of the treatment groups is recommended. Still, findings should inform chemicals and study design elements in future studies with broader samples of firefighters, including the collection of information about individual firefighter activities and personal monitoring.

#### Strengths.

Being able to measure personal exposure appears to be very important given the variability in concentrations and detections of different chemicals between individuals; a stationary air monitor, for example, does not provide the same nuance in data that accounts for the environment, nor do activities and decontamination practices an individual engages with. Silicone passive sampling devices allow us to capture individual exposures of firefighters over time, without significant burden to the firefighter. As demonstrated by this work, and other similar studies, this sampling technology allows for quantitative analysis across a broad chemical space with a single sample [22, 24, 25, 29]. Both advantages make this technology a great alternative to biomonitoring.

The paired study design between on- and off-duty tags allowed for the occupational exposure measurement relative to a personal baseline, with use of fold-changes and binned chemical exposure metrics. While biological samples can be informative for determining exposure and internal dose, the chemical space under investigation is limited and timing exposure-to-sample collection correctly can be challenging [10, 59]. Silicone passive samplers are advantageous in this context because they are easy to collect over a defined period of time, allowing exposure during multiple fire responses and daily activities to be captured.

## CONCLUSIONS

Silicone dog tags were applied as passive samplers to measure personal firefighter exposures while on- and off-duty. Silicone passive sampling allowed for the quantitation of 92 chemical exposures over 30 cumulative days, accounting for a diversity of behaviors and environments across participating firefighters from two different fire departments. Paired on- and off-duty samples were used to parse out exposures that are occupational in source. A total of nineteen chemicals were identified as potential occupational exposures including PAHs, PBDEs, and one VOC. Of these chemical candidates, PBDE 49 and sec-butylbenzene have not previously been reported in the literature to the authors’ knowledge. No association was found between the abundance of the potential occupational exposures with firefighter rank, fire response frequencies, or years in the fire service. However, there are compelling patterns in the data that highlight the impact of individual behaviors and responses to specific fires on exposure, as exemplified by the association between PCB exposure and response of operational firefighters to a specific structural fire. Additionally, environments associated with the fire department influenced firefighter exposure as illustrated with unique and consistent exposure of firefighters at the low call volume department to certain PBDEs while on-duty. Through this study, silicone passive sampling methodology showed great potential in characterizing diverse firefighter chemical exposures, with minimal burden to firefighter participants.

## Supplementary Material

Supplemental Material

## Figures and Tables

**Table 1.** Target analytes for the three analytical methods used: FRs, VOCs, and PCBs. FRs and VOCs are further categorized by structural features.

FR analytes	Polybrominated diphenyl ether (PBDE) congeners	1, 2, 3, 7, 8, 10, 11, 12, 13, 15, 17, 25, 28+33, 30, 32, 35, 37, 47, 49, 66, 71, 75, 77, 99, 100, 116, 118, 119, 138, 153, 154, 166, 181, 183, 190
	Organophosphate flame retardants	triethyl phosphate (TEP), tributyl phosphate (TBP), tris(2-chloroethyl) phosphate (TCEP), tris(1-chloro-2-propanyl) phosphate (TCPP), tris(1,3-dichloro-2-propyl) phosphate (TDCIPP), triphenylphosphate (TPHP)
	Brominated flame retardants	2-ethylhexyl 2,3,4,5-tetrabromobenzoate (EH-TBB), di(2-ethylhexyl) tetrabromophthalate (TBPH)
VOC analytes	Chlorinated benzenes and toluenes	1,2,3-trichlorobenzene, 1,2,4-trichlorobenzene, 1,3-dichlorobenzene, 2-chlorotoluene, 4-chlorotoluene, o-dichlorobenzene, p-dichlorobenzene
	Alkylbenzenes	1,2,3-trimethylbenzene, 1,2,4-trimethylbenzene, 1,3,5-trimethylbenzene, n-butylbenzene, tert-butylbenzene, p-isopropyltoluene, sec-butylbenzene, styrene, xylenes (meta+para)
	Alkanes	n-dodecane, n-hexadecane, n-pentadecane, n-propylbenzene, n-tetradecane
PCB congener analytes		1, 8, 10, 21, 28, 37, 44, 49, 50, 52, 60, 66, 70, 74, 77, 81, 82, 87, 99, 101, 104, 105, 114, 118, 123, 126, 128, 138 +158, 145, 153, 156, 157, 166, 167, 169, 170, 179, 180, 183, 187, 189, 204

Analyte properties and method parameters can be found in the SM. Co-eluting analytes are denoted with a “+”.

**Table 2.** Summary of results for analytes detected in at least six samples.

			High call volume station detections		Low call volume station detections		Paired Difference Sign (On-Off Duty Concentration)			Two-sided Sign Test Result Summary (64 comparisons)				Distribution of paired on- and off-duty ratios							
Chemical Category	Sig	Analyte	Off-duty	On-Duty	Off-Duty	On-Duty	Zero	Negative	Positive	Proportion Positive	p.value	Lower 95% CI	Upper 95% CI	Median Log2FC	Mean Log2FC	SE Log2FC	MedianFC (≤1)	MedianFC (>1)	Google Scholar Results	Verified detection reported (outside of our lab)	Google Scholar Search Terms (“firefighter” AND chemical synonyms including CAS number and common names from SWIFT database)
FR		TPHP	24	30	26	26	0	24	30	0.56	4.97E-01	0.42	0.68	0.18	0.78	0.31	0.66	2.1	19	yes	“firefighter” AND (“115-86-6” OR “triphenyl phosphate” OR “triphenylphosphate” OR “tpp” OR “tphp”)
FR		TDCIPP	24	30	25	26	1	27	26	0.49	1.00E +00	0.36	0.62	−0.053	0.11	0.17	0.63	1.7	62	yes	“firefighter” AND (“13674-87-8” OR “tris(1,3-dichloro-2-propyl) phosphate” OR “fyrol fr 2” OR “fyrol fr-2” OR “tris(1,3-dichloroisopropyl)phosphate” OR “tdcpp” OR “tdcipp”)
FR	***	PBDE 47	21	30	24	26	2	10	42	0.81	9.06E-06	0.68	0.89	1.4	1.8	0.29	0.73	3.3	61	yes	“firefighter” AND (“5436-43-1” OR “4,4’-Oxybis(1,3-dibromobenzene)” OR “2,2’,4,4’-tetrabromodiphenyl ether” OR “pbde 47” OR “BDE 47”)
FR	***	PBDE 99	14	26	16	25	5	7	42	0.86	3.62E-07	0.73	0.93	1.6	2.5	0.38	0.81	12.9	17	yes	“firefighter” AND (“60348-60-9” OR “1,2,4-tribromo-5-(2,4-dibromophenoxy)benzene” OR “2,2’,4,4’,5-pentabromodiphenyl ether” OR “PDBE 99” OR “BDE 99”)
FR		TCPP	11	11	14	15	20	20	14	0.41	3.92E-01	0.26	0.58	0.00	−0.38	0.51	0.90	23.6	45	yes	“firefighter” AND (“26248-87-3” OR “Tris(1-chloro-2-propanyl) phosphate” OR “tcpp” OR “tris(1-chloropropyl) phosphate”)
FR	***	PBDE 100	6	12	10	20	24	5	25	0.83	3.25E-04	0.66	0.93	0.00	1.6	0.35	1.0	17.5	19	yes	“firefighter” AND (“189084-64-8” OR “benzene, 1,3,5-tribromo-2-(2,4-dibromophenoxy)-” OR “2,2’,4,4’,6-pentabromodiphenyl ether” OR “PBDE 100” OR “BDE 100”)
FR		TCEP	7	13	5	12	25	11	18	0.62	2.65E-01	0.44	0.77	0.00	0.99	0.47	1.0	36.0	56	yes	“firefighter” AND (“115-96-8” OR “tris(2-chloroethyl) phosphate” OR “fyrol cef” OR “tri(2-chloroethyl)phosphate” OR “tris(beta-chloroethyl) phosphate” OR “TCEP”)
FR		Tributyl phosphate	9	14	12	9	26	11	17	0.61	3.45E-01	0.42	0.76	0.00	0.19	0.48	1.0	2.3	34	only 1 (biomarker of)	“firefighter” AND (“126-73-8” OR “tributyl phosphate” OR “tri-n-butyl phosphate” OR “tri-n-butylphosphate” OR “tributylphosphate”)
FR		EH-TBB	9	12	6	8	31	7	16	0.70	9.31E-02	0.49	0.84	0.00	0.64	0.41	1.0	13.2	68	yes	“firefighter” AND (“183658-27-7” OR “benzoic acid, 2,3,4,5-tetrabromo-, 2-ethylhexyl ester” OR “2-ethylhexyl 2,3,4,5-tetrabromobenzoate” OR “TBB” OR “EH-TBB”)
FR		Triethyl phosphate	3	3	11	14	33	9	12	0.57	6.64E-01	0.37	0.76	0.00	0.16	0.32	1.0	13.8	24	yes	“firefighter” AND (“78-40-0” OR “triethyl phosphate” OR “triethylphosphate” OR “tris(ethyl) phosphate”)
FR		PBDE 154	2	3	2	11	41	3	10	0.77	9.23E-02	0.50	0.92	0.00	0.76	0.23	1.0	21.6	25	yes	“firefighter” AND (“207122-15-4” OR “benzene, 1,3,5-tribromo-2-(2,4,5-tribromophenoxy)-” OR “2,2’,4,4’,5,6’-hexabromodiphenyl ether” OR “PBDE 154” OR “BDE 154”)
FR	**	PBDE 28 + 33	1	1	0	12	41	1	12	0.92	3.42E-03	0.67	1.0	0.00	1.0	0.26	1.0	22.0	21,0	yes,no	“firefighter” AND (“41318-75-6” OR “benzene, 2,4-dibromo-1-(4-bromophenoxy)-” OR “2,4,4’-Tribromodiphenyl ether” OR “PBDE 28” OR “BDE 28”); “firefighter” AND (“147217-78-5” OR “2’,3,4-tribromodiphenyl ether” OR “1,2-dibromo-4-(2-bromophenoxy)benzene” OR “PBDE 33” OR “BDE 33”)
FR		PBDE 153	2	3	1	8	43	3	8	0.73	2.27E-01	0.43	0.9	0.00	0.69	0.24	1.0	26.0	26	yes	“firefighter” AND (“68631-49-2” OR “benzene, 1,1’-oxybis(2,4,5-tribromo-” OR “2,2’,4,4’,5,5’-hexabromodiphenyl ether” OR “PBDE 153” OR “BDE 153”)
FR	*	PBDE 49	1	1	0	8	45	1	8	0.89	3.91E-02	0.57	0.99	0.00	0.66	0.23	1.0	24.6	8	no	“firefighter” AND (“243982-82-3” OR “1,4-dibromo-2-(2,4-dibromophenoxy)benzene” OR “2,2’,4,5’-tetrabromodiphenyl ether” OR “PBDE 49” OR “BDE 49”)
FR		PBDE 17	1	1	0	5	48	1	5	0.83	2.19E-01	0.44	0.99	0.00	0.31	0.14	1.0	8.7	10	no	“firefighter” AND (“147217-75-2” OR “2,2’,4-Tribromodiphenyl ether” OR “PBDE 17” OR “BDE 17” OR “2,4-Dibromo-1-(2- bromophenoxy) benzene”)
PAH		1-methylnaphthalene	27	29	27	27	0	24	30	0.56	4.97E-01	0.42	0.68	0.16	0.09	0.22	0.70	1.8	47	yes	“firefighter” AND (“90-12-0” OR “1-methylnaphthalene” OR “alpha-methylnaphthalene”)
PAH	**	2,6-dimethylnaphthalene	27	29	27	27	0	16	38	0.70	3.84E-03	0.57	0.81	0.25	0.38	0.17	0.84	1.5	6	only 1	“firefighter” AND (“581-42-0” OR “2,6-dimethylnaphthalene”)
PAH		2-ethylnaphthalene	26	29	26	26	0	20	34	0.63	7.59E-02	0.50	0.75	0.19	0.52	0.25	0.82	2.0	7	no	“firefighter” AND (“939-27-5” OR “naphthalene, 2-ethyl-” OR “2-ethylnaphthalene”)
PAH		2-methylanthracene	25	29	27	27	0	22	32	0.59	2.20E-01	0.46	0.71	0.14	0.48	0.25	0.77	1.5	7	no	“firefighter” AND (“613-12-7” OR “2-methylanthracene”)
PAH		2-methylnaphthalene	27	29	27	27	0	23	31	0.57	3.41E-01	0.44	0.70	0.12	0.075	0.21	0.67	1.8	50	yes	“firefighter” AND (“91-57-6” OR “2-methylnaphthalene” OR “beta-methylnaphthalene”)
PAH		2-methylphenanthrene	26	30	27	27	0	24	30	0.56	4.97E-01	0.42	0.68	0.099	0.40	0.21	0.83	1.5	10	yes	“firefighter” AND (“2531-84-2” OR “2-methylphenanthrene”)
PAH		dibenzothiophene	27	29	27	27	0	26	28	0.52	8.92E-01	0.39	0.65	0.048	−0.025	0.17	0.76	1.4	16	only 1	“firefighter” AND (“132-65-0” OR “dibenzothiophene” OR “dibenzo(b,d)thiophene”)
PAH	***	fluoranthene	26	30	26	27	0	8	46	0.85	1.38E-07	0.73	0.92	0.56	0.87	0.19	0.82	1.7	237	yes	“firefighter” AND (“206-44-0” OR “fluoranthene”)
		naphthalene	27	29	27	27	0	32	22	0.41	2.20E-01	0.29	0.54	−0.34	−0.22	0.20	0.60	1.7	509	yes	“firefighter” AND (“91-20-3” OR “naphthalene”)
PAH	**	phenanthrene	27	30	27	27	0	17	37	0.69	9.07E-03	0.55	0.79	0.27	0.34	0.11	0.84	1.4	305	yes	“firefighter” AND (“85-01-8” OR “phenanthrene”)
PAH	**	fluorene	26	30	24	27	1	17	36	0.68	1.27E-02	0.55	0.79	0.36	0.83	0.24	0.84	1.7	195	yes	“firefighter” AND (“86-73-7” OR “fluorene” OR “9h-fluorene”)
PAH	***	pyrene	26	30	27	27	1	10	43	0.81	5.55E-06	0.69	0.89	0.5	0.65	0.16	0.90	1.6	576	yes	“firefighter” AND (“129-00-0” OR “pyrene”)
PAH	***	retene	26	30	27	27	1	10	43	0.81	5.55E-06	0.69	0.89	0.5	0.65	0.13	0.90	1.6	41	yes	“firefighter” AND (“483-65-8” OR “retene” OR “1-methyl-7-isopropylphenanthrene” OR “7-isopropyl-1-methylphenanthrene”)
PAH		1,2-dimethylnaphthalene	21	25	20	23	6	19	29	0.60	1.93E-01	0.46	0.73	0.064	0.74	0.36	0.76	2.0	5	only 1	“firefighter” AND (“573-98-8” OR “1,2-dimethylnaphthalene” OR “1,2-dimethylnaphthalene”)
PAH		1,4-dimethylnaphthalene	21	24	23	22	8	16	30	0.65	5.41E-02	0.51	0.77	0.081	0.14	0.21	0.91	1.5	7	only 1	“firefighter” AND (“571-58-4” OR “1,4-dimethylnaphthalene”)
PAH	*	1,5-dimethylnaphthalene	20	22	22	23	9	15	30	0.67	3.57E-02	0.52	0.79	0.18	0.51	0.20	0.90	2.6	7	only 1	“firefighter” AND (“571-61-9” OR “1,5-dimethylnaphthalene”)
PAH	*	1-methylpyrene	18	26	17	19	9	14	31	0.69	1.61E-02	0.54	0.80	0.16	0.80	0.32	0.96	1.6	11	yes	“firefighter” AND (“2381-21-7” OR “1-methylpyrene”)
PAH		3,6-dimethylphenanthrene	18	20	19	18	11	17	26	0.60	2.22E-01	0.46	0.74	0.00	0.25	0.30	0.76	2.1	10	only 1	“firefighter” AND (“1576-67-6” OR “3,6-dimethylphenanthrene”)
PAH	***	benzo[b]fluoranthene	16	24	14	16	12	7	35	0.83	1.51E-05	0.69	0.92	0.30	1.1	0.35	1.0	1.5	185	yes	“firefighter” AND (“205-99-2” OR “benzo(b)fluoranthene” OR “3,4-benzofluoranthene” OR “benzo[b]fluoranthene”)
PAH	*	anthracene	15	22	10	9	18	11	25	0.69	2.88E-02	0.53	0.82	0.00	0.64	0.33	1.0	3.7	313	yes	“firefighter” AND (“120-12-7” OR “anthracene” OR “anthracen” OR “anthracin” OR “paranaphthalene”)
PAH	***	benzo[e]pyrene	11	21	5	13	19	6	29	0.83	1.17E-04	0.67	0.92	0.23	1.3	0.26	1.0	9.1	75	yes	“firefighter” AND (“192-97-2” OR “benzo(e)pyrene” OR “1,2-benzopyrene” OR “1,2-benzpyrene” OR “benz(e)pyrene” OR “benzo[e]pyrene”)
PAH	***	benzo[k]fluoranthene	10	19	5	11	23	6	25	0.81	8.78E-04	0.64	0.91	0.00	1.3	0.37	1.0	27.9	167	yes	“firefighter” AND (“207-08-9” OR “benzo(k)fluoranthene” OR “benz(k)fluoranthene” OR “benzo[k]fluoranthene”)
PAH		acenaphthylene	2	10	10	5	32	10	12	0.55	8.32E-01	0.35	0.73	0.00	0.065	0.19	1.0	3.2	146	yes	“firefighter” AND (“208-96-8” OR “acenaphthylene” OR “acenaphthalene” OR “Cyclopenta(de)naphthalene”)
PAH	***	chrysene	2	13	1	4	38	1	15	0.94	5.19E-04	0.72	1.0	0.00	1.00	0.25	1.0	13.2	238	yes	“firefighter” AND (“218-01-9” OR “chrysene” OR “1,2-benzophenanthrene” OR “benzo(a)phenanthrene”)
PAH	***	triphenylene	3	12	1	2	41	0	13	1.0	2.44E-04	0.77	1.0	0.00	0.87	0.26	1.0	34.2	22	yes	“firefighter” AND (“217-59-4” OR “triphenylene” OR “9,10-benzophenanthrene” OR “isochrysene” OR “triphenylene”)
PAH	*	benz[a]anthracene	4	8	0	2	44	1	9	0.90	2.15E-02	0.60	0.99	0.00	0.50	0.22	1.0	21.1	124	yes	“firefighter” AND (“56-55-3” OR “benz(a)anthracene” OR “1,2-benz(a)anthracene” OR “1,2-benzanthracene” OR “1,2-benzoanthracene” OR “benzanthracene” OR “benzoanthracene” OR “tetraphene”)
PAH		benzo[a]pyrene	3	8	1	2	44	2	8	0.80	1.09E-01	0.49	0.94	0.00	0.24	0.10	1.0	2.4	174	yes	“firefighter” AND (“50-32-8” OR “benzo(a)pyrene” OR “1,2-benzpyrene” OR “3,4-benz(a)pyrene” or “b(a)p”)
PAH	*	benzo[j]fluoranthene	2	10	0	1	44	1	9	0.90	2.15E-02	0.6	0.99	0.00	0.26	0.12	1.0	2.2	63	yes	“firefighter” AND (“205-82-3” OR “benzo(j)fluoranthene” OR “benz(j)fluoranthene” OR “benzo[j]fluoranthene”)
PAH		acenaphthene	1	6	1	0	48	1	5	0.83	2.19E-01	0.44	0.99	0.00	0.42	0.30	1.0	99.2	150	yes	“firefighter” AND (“83-32-9” OR “acenaphthene” OR “1,2-Dihydroacenaphthylene” OR “1,8-Ethylenenaphthalene”)
PAH	*	benzo[a]fluorene	2	6	0	0	48	0	6	1.0	3.13E-02	0.61	1.0	0.00	0.26	0.12	1.0	6.6	3	only 1	“firefighter” AND (“238-84-6” OR “benzo(a)fluorene” OR “1,2-benzofluorene” OR “11h-benzo(a)fluorene” OR “chrysofluorene”)
PCB		PCB 153	4	8	3	4	41	6	7	0.54	1.00E + 00	0.29	0.77	0.00	0.47	0.38	1.0	126	25	yes	“firefighter” AND (“35065-27-1” OR “2,4,5,2’,4’,5’-hexachlorobiphenyl” OR “2,2’,4,4’,5,5’-hexachlorobiphenyl” OR “pcb 153”)
VOC		n-Tetradecane	19	22	24	24	6	21	27	0.56	4.71E-01	0.42	0.69	0.028	0.21	0.54	0.65	1.8	31	only 1 (tentative)	“firefighter” AND (“629-59-4” OR “n-tetradecane” OR “tetradecane”)
VOC		n-Dodecane	14	20	23	23	8	19	27	0.59	3.02E-01	0.44	0.72	0.014	0.43	0.58	0.81	2.0	61	yes	“firefighter” AND (“112-40-3” OR “dodecane” OR “n-dodecane”)
VOC		n-Pentadecane	16	20	23	22	10	19	25	0.57	4.51E-01	0.42	0.70	0.00	0.56	0.65	0.71	2.1	15	only 1 (tentative)	“firefighter” AND (“629-62-9” OR “n-pentadecane” OR “pentadecane”)
VOC		1,2,3-Trimethylbenzene	17	21	22	22	13	17	24	0.59	3.49E-01	0.43	0.72	0.00	0.50	0.36	0.94	1.7	3	no	“firefighter” AND (“526-73-8” OR “1,2,3-trimethylbenzene” OR “hemimellitene” OR “hemimellitene”)
VOC		1,3,5-Trimethylbenzene	17	20	23	21	13	19	22	0.54	7.55E-01	0.39	0.68	0.00	−0.063	0.45	0.93	1.8	5	no	“firefighter” AND (“108-67-8” OR “mesitylene” OR “1,3,5-trimethylbenzene” OR “sym-trimethylbenzene” OR “trimethylbenzol” OR “mesitylene”)
VOC	*	p-Isopropyltoluene	11	11	16	15	23	9	22	0.71	2.94E-02	0.53	0.84	0.00	0.0021	0.41	1.0	2.1	24	yes	“firefighter” AND (“99-87-6” OR “p-cymene” OR “1-isopropyl-4-methylbenzene” OR “4-cymene” OR “4-isopropyltoluene” OR “p-isopropyltoluene” OR “paracymene” OR “paracymene”)
VOC		1,2,4-Trimethylbenzene	14	15	9	9	30	13	11	0.46	8.39E-01	0.28	0.65	0.00	−0.20	0.28	1.0	1.6	31	yes	“firefighter” AND (“95-63-6” OR “1,2,4-trimethylbenzene” OR “1,3,4-trimethylbenzene” OR “pseudocumene” OR “pseudocumol” OR “pseudocumene”)
VOC		Xylenes(m + p)	4	10	9	9	32	6	16	0.73	5.25E-02	0.52	0.87	0.00	1.0	0.61	1.0	184	53,89	yes	“firefighter” AND (“108-38-3” OR “3-xylene” OR “1,3-dimethylbenzene” OR “1,3-xylene” OR “m-xylene” OR “m-xylol” OR “3-xylene” OR “meta-xylene”); “firefighter” AND (“106-42-3” OR “4-xylene” OR “1,4-dimethylbenzene” OR “1,4-xylene” OR “p-dimethylbenzene” OR “p-xylene” OR “p-xylol” OR “4-xylene” OR “para-xylene”)
VOC	*	n-Butylbenzene	3	6	8	9	39	3	12	0.80	3.52E-02	0.55	0.93	0.00	0.22	0.27	1.0	1.9	12	no	“firefighter” AND (“104-51-8” OR “n-butylbenzene” OR “1-phenylbutane” OR “butylbenzene”)
VOC	*	sec-Butylbenzene	1	5	6	11	39	3	12	0.80	3.52E-02	0.55	0.93	0.00	1.0	0.40	1.0	162	4	no	“firefighter” AND (“135-98-8” OR “sec-butylbenzene” OR “2-phenylbutane” OR “(1-methylpropyl)benzene”)
VOC		p-Dichlorobenzene	0	1	10	11	40	5	9	0.64	4.24E-01	0.39	0.84	0.00	0.072	0.20	1.0	1.9	52	yes	“firefighter” AND (“106-46-7” OR “1,4-dichlorobenzene” OR “p-dichlorobenzene” OR “paradichlorobenzene”)
VOC		Styrene	5	5	1	2	45	4	5	0.56	1.00E + 00	0.27	0.81	0.00	0.28	0.40	1.0	398	691	yes	“firefighter” AND (“100-42-5” OR “styrene” OR “cinnamene” OR “ethenylbenzene” OR “phenethylene” OR “phenylethene” OR “styrol” OR “styrole” OR “styrolene” OR “vinyl benzene” OR “vinylbenzene”)
VOC		n-Propylbenzene	3	7	1	2	45	2	7	0.78	1.80E-01	0.45	0.94	0.00	0.71	0.45	1.0	1080	27	yes	“firefighter” AND (“103-65-1” OR “n-propylbenzene” OR “1-phenylpropane” OR “isocumene” OR “phenylpropane” OR “propylbenzene”)

The “Sig” column denotes the significance of the sign test result

* <0.05,

** <0.01,

*** <0.000781 (Bonferroni corrected threshold)).

## Data Availability

The datasets generated during the current study are not publicly available due to the involvement of human subjects but are available from the corresponding author on reasonable request.
