# Supplementary material for: Addressing the need for individual-level exposure monitoring for firefighters using silicone samplers
Source: J Expo Sci Environ Epidemiol. 2024 Jul 20;35(2):180–95. doi: 10.1038/s41370-024-00700-y (PMC11743823; doi:10.1038/s41370-024-00700-y)
Supplement: Supplementary file 1 — Supplementary Material [file 41370_2024_700_MOESM1_ESM.docx]

**Supplementary Material**

**Title:** **Addressing the need for individual-level exposure monitoring for firefighters using silicone samplers**

**Authors: Emily M Bonner ^1^, Carolyn M Poutasse ^2^, Christopher K Haddock ^3^, Walker S C Poston ^3^, Sara A Jahnke ^3^, Lane G Tidwell ^1^, Kim A Anderson ^1,4^**

1. Department of Environmental and Molecular Toxicology, Oregon State University, Corvallis, OR 97331, United States.
2. California Air Resources Board, Sacramento, CA 95814, United States.
3. Center for Fire, Rescue, and EMS Health Research, National Development and Research Institutes, Inc. (NDRI)-USA, Leawood, KS 66224, United States.
4. Corresponding author, Electronic address: kim.anderson@oregonstate.edu.

**The statements and conclusions expressed in this article are those of the authors and do not necessarily reflect the views or positions of the California Air Resources Board*.

**Table of Contents**

[S1. Methods S1](#_Toc166615163)

[S1.1 Extraction S1](#_Toc166615164)

[S1.2 Instrument Analysis S1](#_Toc166615165)

[S1.3 QA/QC S1](#_Toc166615166)

[S1.3.1 Flame Retardants S2](#_Toc166615167)

[S1.3.2 VOCs S2](#_Toc166615168)

[S1.3.3 PCBs S2](#_Toc166615169)

[S2. Instrumental method parameters and analyte information S3](#_Toc166615170)

[S2.1 Flame Retardants S3](#_Toc166615171)

[S2.2 VOCs S5](#_Toc166615172)

[S2.3 PCBs S7](#_Toc166615173)

[S3. Additional notes on quantitation S9](#_Toc166615174)

[S4. Data tables S10](#_Toc166615175)

[S5. Supplementary figures S13](#_Toc166615176)

[References S19](#_Toc166615177)

# **S1. Methods**

## ***S1.1 Extraction***

Deuterated analytes were added to samples as extraction recovery surrogates prior to extracting the silicone samples in two 50 mL volumes of ethyl acetate, which were combined. The sample extracts were concentrated to one mL under nitrogen (TurboVap L, Biotage, Charlotte, NC, USA; RapidVap, LabConco, Kansas City, MO, USA; N-EVAP 111, Organomation Associates, Berlin, MA, USA). Solid phase extraction with acetonitrile was used to further process 100 µL aliquots of each sample (Cleneart S C18, Agela Technologies, Torrance, CA, USA)(1). Finally, samples were solvent-exchanged into iso-octane (OA-SYS N-EVAP 111) and stored at -4°C until instrument analysis. Deuterated standard recoveries are reported in section “S1.3 QA/QC.”

Of the 21 VOC target analytes, seven chemicals are known to have reduced recovery levels from silicone samples following liquid and solid phase extraction (between 30 and 50% recovery) based on method validation experiments. This is noted, along with method information for all analytes and whether a background correction was needed for the analyte based on detections in QC samples in **Table S3**.

## ***S1.2 Instrument Analysis***

Each analyte in all instrument methods was calibrated with linear curves spanning 1 pg/µL to 1 ng/µL with at least five points and correlations ≥0.99. To determine the limits of detection (LODs) and limits of quantitation (LOQs) for each analyte, the lowest standard with a 15:1 signal-to-noise ratio was run seven times. The resulting standard deviation was used to calculate a 99% confidence interval with the Student’s t-value and appropriate degree of freedom. LOQs were five times higher than the LODs. Method specific details can be found below.

**Flame Retardants:** Forty-three flame retardant analytes, including 35 PDBEs, six OPFRs, and two BFRs were quantified using an Agilent 7890A GC and Agilent 5975C MS. PBDEs 28 and 33 were quantified together due to coelution of the isomers. Limits of detection range from 0.7 pg/μL - 25 pg/μL (**Table S6**). Full validation of this method is published in Kile et al., 2016 (2).

**Volatile Organic Compounds:** Twenty-one VOC analytes were quantified using an Agilent 7890A GC and Agilent 5975C MS (**Table S3**). Meta- and ortho-xylene isomers were quantified together due to coelution of the isomers.

**Polychlorinated Biphenyls:** GC/MS (Agilent 8890 gas chromatograph; Agilent 5977B mass spectrometer) was used to quantify 43 PCB congeners (PCBs 138 and 158 co-elute; a sum concentration of the two congeners was reported). Detection limits were determined routinely to be < 5 pg/μL (**Table S1**).

## ***S1.3 QA/QC***

Continuing calibration verifications (CCVs) were analyzed before and after each batch, and after a maximum of 13 samples. Instrument blanks were run after each CCV and after a maximum of five samples. At a minimum, the following QC samples were analyzed on each instrument method: lab processing blanks (n=2), trip blanks (n=2), post-deployment cleaning blanks (n=3), a solid phase extraction blank, and an extraction reagent blank. Trip blanks are non-deployed dog tags placed in sealed PTFE bags during wristband deployment and mailed via the United States Postal Service from Kansas City to Oregon State University. The only flame retardant detected in any QC sample was triphenyl phosphate (24 pg/µL), which was only found in one post-deployment cleaning blank, corresponding to the processing of a single sample. Background corrections for VOCs in samples were performed using concentrations found in the corresponding laboratory processing blank (n=2), which were exposed to all laboratory processes (**Table S5**). No PCBs were detected in these blanks.

### *S1.3.1 Flame Retardants*

Two duplicate sample aliquots were analyzed for flame retardants, with an average relative percent difference in concentration of 9% for all detected analytes. Two matrix spike duplicates analyzed for flame retardants had a mean recovery of 85% for all analytes. 87% of analytes were quantified within 30% of the true value across CCVs (n=18), with a mean extraction surrogate recovery of 117% and a mean relative standard deviation of 17% for the internal standard response.

### *S1.3.2 VOCs*

For the VOC analysis, duplicate samples had an average relative percent difference in concentration of 23% for all detected VOCs. Two matrix spike duplicates analyzed on the VOC method showed an average percent recovery for analytes of 72%. On average, 97% of target analytes were quantified within 30% of the true standard concentration in the CCVs. CCVs had a mean extraction surrogate recovery of 41% and a mean relative standard deviation of 45% for the internal standard response.

### *S1.3.3 PCBs*

Duplicate sample aliquots (n=4) analyzed on the PCB method had an average relative percent difference in concentration of 8.1% for all detected PCBs. Matrix spike duplicates analyzed with the PCB method (n=2) had a mean percent recovery for detected analytes of 130%, ranging from 84% (PCB 180) to 140% (PCB 138+158). On average, 92% of analytes were quantified within 25% of the true value across CCVs, with a mean extraction surrogate recovery of 140% and a mean relative standard deviation of 69% for the internal standard response.

# **S2. Instrumental method parameters and analyte information**

## ***S2.1 Flame Retardants***

**Table S1.** Flame Retardant GC-EI/MS method and validation parameters. Superscripts indicate what compound is used for internal calibration.

| **Flame Retardant Compound** | | | **GC/MS Parameters** | | | | |
| --- | --- | --- | --- | --- | --- | --- | --- |
|  | **PBDE congener number or abbreviation** | **CAS Number** | **Retention time (min)** | **SIM Ion (m/z)** | **Qual Ions (m/z)** | **LOD (ng/mL)^f^** | **LOQ (ng/mL)^f^** |
| **Polybrominated diphenyl ethers (PBDE)** | | | | | | | |
| 2-Bromodiphenyl ether ^d^ | PBDE 1 | 7025-06-1 | 11.62 | 169 | 248, 250 | 2.03 | 10.1 |
| 3-Bromodiphenyl ether ^d^ | PBDE 2 | 6876-00-2 | 11.82 | 248 | 250, 141 | 1.66 | 8.3 |
| 4-bromodiphenyl ether ^d^ | PBDE 3 | 101-55-3 | 12.02 | 248 | 250, 141 | 1.86 | 9.3 |
| di(4-Bromophenyl)ether ^d^ | PBDE 10 | 51930-04-2 | 13.95 | 168 | 328, 326 | 1.91 | 9.6 |
| 3,4’-Dibromodiphenyl ether ^d^ | PBDE 7 | 171977-44-9 | 14.56 | 168 | 328, 330 | 1.56 | 7.8 |
| 3,3’-Dibromodiphenyl ether ^d^ | PBDE 11 | 6903-63-5 | 14.85 | 328 | 326, 167 | 1.62 | 8.1 |
| 2,4’-Dibromodiphenyl ether ^d^ | PBDE 8 | 147217-71-8 | 14.88 | 168 | 328, 326 | 1.51 | 7.5 |
| 1,2-Dibromodiphenyl ether ^d^ | PBDE 12 | 189084-59-1 | 15.04 | 328 | 326, 330 | 1.59 | 8.0 |
| 3,4’-Dibromodiphenyl ether ^d^ | PBDE 13 | 83694-71-7 | 15.09 | 328 | 326, 330 | 1.05 | 5.2 |
| 4,4’-Dibromodiphenyl ether ^d^ | PBDE 15 | 2050-47-7 | 15.32 | 328 | 326, 330 | 0.93 | 4.6 |
| 2,4,6-Tribromodiphenyl ether ^d^ | PBDE 30 | 155999-95-4 | 16.32 | 246 | 406, 408 | 1.53 | 7.6 |
| 2,4’,6-Tribromodiphenyl ether ^d^ | PBDE 32 | 189084-60-4 | 16.99 | 246 | 406, 408 | 1.67 | 8.4 |
| 2,2’,4-Tribromodiphenyl ether ^d^ | PBDE 17 | 147217-75-2 | 17.14 | 246 | 406, 408 | 1.65 | 8.3 |
| 2,3’,4-Tribromodiphenyl ether ^d^ | PBDE 25 | 147217-77-4 | 17.20 | 246 | 406, 408 | 1.23 | 6.1 |
| 2,4,4’-Tribromodiphenyl ether, 2’,3,4-Tribromodiphenyl ether ^d^ | PBDE 28+33 | 41318-75-6; 147217-78-5 | 17.44 | 246 | 406, 408 | 1.12 | 5.6 |
| 3,3’,4-Tribromodiphenyl ether ^d^ | PBDE 35 | 147217-80-9 | 17.62 | 406 | 408, 410 | 3.19 | 15.9 |
| 3,4,4’-Tribromodiphenyl ether ^d^ | PBDE 37 | 147217-81-0 | 17.83 | 406 | 408, 404 | 0.72 | 3.6 |
| 2,4,4’,6-Tetrabromodiphenyl ether ^g^ | PBDE 75 | 189084-63-7 | 18.67 | 326 | 486, 488 | 1.88 | 9.4 |
| 2,2’,4,5’-Tetrabromodiphenyl ether ^g^ | PBDE 49 | 243982-82-3 | 18.77 | 326 | 486, 488 | 1.91 | 9.5 |
| 2,3’,4’,6-Tetrabromodiphenyl ether ^g^ | PBDE 71 | 189084-62-6 | 18.84 | 326 | 486, 484 | 1.37 | 6.8 |
| 2,2’,4,4’-Tetrabromodiphenyl ether ^g^ | PBDE 47 | 5436-43-1 | 19.00 | 326 | 486, 484 | 2.08 | 10.4 |
| 2,3’,4,4’-Tetrabromodiphenyl ether ^g^ | PBDE 66 | 189084-61-5 | 19.22 | 326 | 486, 484 | 2.54 | 12.7 |
| 3,3’,4,4’-Tetrabromodiphenyl ether ^g^ | PBDE 77 | 93703-48-1 | 19.53 | 486 | 484, 488 | 0.84 | 4.2 |
| 2,2’,4,4’,6-Pentabromodiphenyl ether ^g^ | PBDE 100 | 189084-64-8 | 19.96 | 404 | 566, 564 | 2.39 | 12.0 |
| 2,3’,4,4’,6-Pentabromodiphenyl ether ^g^ | PBDE 119 | 189084-66-0 | 20.09 | 406 | 564, 566 | 1.64 | 8.2 |
| 2,2’,4,4’,5-Pentabromodiphenyl ether ^g^ | PBDE 99 | 60348-60-9 | 20.26 | 404 | 564, 566 | 2.32 | 11.6 |
| 2,3,4,5,6-Pentabromodiphenyl ether ^g^ | PBDE 116 | 189084-65-9 | 20.42 | 406 | 564, 566 | 2.17 | 10.8 |
| 2,3’,4,4’,5-Pentabromodiphenyl ether ^g^ | PBDE 118 | 446254-80-4 | 20.54 | 404 | 564, 566 | 2.30 | 11.5 |
| 2,2’,4,4’,5,6’-Hexabromodiphenyl ether ^g^ | PBDE 154 | 207122-15-4 | 21.13 | 484 | 644, 642 | 1.61 | 8.1 |
| 2,2’,4,4’,5,5’-Hexabromodiphenyl ether ^g^ | PBDE 153 | 68631-49-2 | 21.67 | 484 | 644, 642 | 1.33 | 6.7 |
| 2,2’,3,4,4’,5’-Hexabromodiphenyl ether ^g^ | PBDE 138 | 182677-30-1 | 22.51 | 484 | 644, 642 | 2.04 | 10.2 |
| 2,3,4,4’,5,6-Hexabromodiphenyl ether ^g^ | PBDE 166 | 189084-58-0 | 22.64 | 484 | 646, 644 | 1.34 | 6.7 |
| 2,2',3,4,4',5',6-Heptabromodiphenyl ether ^g^ | PBDE 183 | 207122-16-5 | 23.33 | 564 | 722, 723 | 20.83 | 104.1 |
| 2,2’,3,4,4’,5,6-Heptabromodiphenyl ether ^g^ | PBDE 181 | 189084-67-1 | 25.11 | 564 | 723, 722 | 24.23 | 121.1 |
| 2,3,3’,4,4’,5,6-Heptabromodiphenyl ether ^g^ | PBDE 190 | 189084-68-1 | 25.42 | 562 | 722, 724 | 13.72 | 68.6 |
| **Organophosphate flame retardants (OPFRs)** | | | | | | | |
| Triethyl phosphate ^b^ | TEP | 78-40-0 | 4.53 | 99 | 155, 81 | 5.3 | 26.5 |
| Tributyl phosphate ^b^ | TBP | 126-73-8 | 11.29 | 99 | 155, 211 | 3.2 | 16 |
| Tris(2-chloroethyl) phosphate ^c^ | TCEP | 115-96-8 | 12.61 | 249 | 251, 205 | 15.73 | 78.6 |
| Tris(1-chloro-2-propanyl) phosphate ^b^ | TCPP | 26248-87-3 | 12.95, 13.11 | 157 | 277, 279 | 24.55 | 122.7 |
| Tris(1,3-dichloro-2-propyl) phosphate ^f^ | TCIDPP | 13674-87-8 | 17.77 | 381 | 383, 379 | 23.99 | 119.9 |
| Triphenylphosphate ^f^ | TPHP | 115-86-6 | 18.27 | 326 | 325, 327 | 1.15 | 5.7 |
| **Brominated flame retardants (BFRs)** | | | | | | | |
| 2-Ethylhexyl 2,3,4,5-Tetrabromobenzoate ^g^ | EH-TBB | 183658-27-7 | 20.25 | 112 | 421, 419 | 2.77 | 13.9 |
| Di(2-ethylhexyl)tetrabromophthalate ^f^ | TBPH | 26040-51-7 | 25.25 | 112 | 464, 467 | 12.42 | 62.1 |
| **Internal Standards** | | | | | | | |
| 6-fluoro-2,2’,4,4’-Tetrabromodiphenyl ether (a) | 6-FBDE-47 (IS) | 876310-23-5 | 18.56 | 504 | 344, 502 | N/A | N/A |
| Tributyl phosphate-d27^a^ (b) | TBP-d27 (SS) | 61196-27-7 | 11.08 | 103 | 167, 231 | 1.00 | 5.0 |
| Tris(2-chloroethyl) phosphate-d12^a^ (c) | TCEP-d12 (SS) | 1276500-47-0 | 12.49 | 261 | 263, 213 | 1.00 | 5.0 |
| 2-fluoro-4,4’-Dibromodiphenyl ether^a^ (d) | 2-FBDE-15(SS) | -- | 14.94 | 341 | 339, 243 | 1.00 | 5.0 |
| 2,2',4,4',6-pentachlorobiphenyl^a^ (e) | PCB 100 (SS) | 39485-83-1 | -- | 326 | 254, 324 | 1.00 | 5.0 |
| Triphenylphosphate-d15^a^ (f) | TPHP-d15 (SS) | 1173020-30-8 | 18.21 | 341 | 339, 243 | 1.00 | 5.0 |
| 3-fluoro-2,2’,4,4’,6-Pentabromodiphenyl ether^a^ (g) | 3-FBDE-100 (SS) | 887401-80-1 | 19.92 | 422 | 424, 582 | 1.00 | 5.0 |
| Benzo[a]pyrene-d12^a^ (h) | B[a]P-d12 (SS) | 63466-71-7 | -- | 264 | 265, 260 | 1.00 | 5.0 |
| 3-fluoro-2,2’,4,4’,5,5’-Hexabromodiphenyl ether^a^ (i) | 3-FBDE-153 (SS) | -- | 21.91 | 502 | 504, 661 | 1.00 | 5.0 |

**Table S2**: Flame Retardant GC/MS Instrument Control Parameters

| **Carrier Gas:** Helium (99.99%) | | | | | |
| --- | --- | --- | --- | --- | --- |
| **Oven Program** | | | | **Inlet Parameters** | |
| **Time (min) and temperature program (^o^C)** | | | | **Mode: Pulsed Splitless** | |
| 90°C, 1.25 min hold | | | | Temp | 290°C |
| 10°C/min to 240°C, 0 min hold | | | | Septum Purge | 3mL/min |
| 20°C/min to 310°C, 10 min hold | | | | Injector Pulse Pressure | 35 psi until 0.5 min |
| 29.75min Total Run Time | | | | Purge to Split Vent | 35 mL/min at 1.00min |
| **Other Settings** | | | | | |
| **Heating elements** | | | | **Value (°C)** | **Maximum** |
| Thermal Auxiliary | | | | 300 | - |
| MS Source | | | | 250 | 280 |
| MS Quad | | | | 150 | 200 |
| **Sim Group Parameters (Acquisition Mode=SIM/Scan)** | | | | | |
| **Group #** | **Start Time/Dwell** | | **Ions** | | |
| 1 | 0 min/20 | | 141.00, 152.00, 169.00, 232.00, 234.00, 248.00, 250.00 | | |
| 2 | 12.80 min/20 | | 125.00, 157.00, 205.00, 249.00, 251.00, 277.00, 279.00, 281.00 | | |
| 3 | 13.80 min/20 | | 168.00, 246.00, 326.00, 328.00, 330.00, 406.00, 408.00 | | |
| 4 | 17.10 min/20 | | 246.00, 325.00, 326.00, 327.00, 379.00, 381.00, 383.00, 404.00, 406.00, 408.00, 410.00 | | |
| 5 | 18.90 min/20 | | 326.00, 484.00, 486.00, 488.00 | | |
| 6 | 20.10 min/20 | | 112.00, 404.00, 406.00, 419.00, 421.00, 564.00, 566.00, 580.00, 582.00, 584.00 | | |
| 7 | 21.25 min/40 | | 260.00, 264.00, 265.00, 484.00, 642.00, 644.00, 646.00 | | |
| 8 | 24.00 min/40 | | 112.00, 465.00, 467.00, 562.00, 564.00, 722.00, 724.00 | | |
| SCAN mass Range | | | | 50-800 | |
| **Injector Settings** | | | | | |
| Injector Volume (μL) | | 1 | | Solvent A and B | 8 µL wash volumes |
| Draw Speed (μL/min) | | 300 | | Solvent A and B pre-injection | 2 washes |
| Eject Speed (μL/min) | | 6000 | | Solvent A and B post-injection | 3 washes |
| **Column Settings & Specifications** | | | | | |
| Column Type | | J&W Scientific, DB5-MS | | | |
| **Dimensions** | | | | | |
| Film (μm) | | 0.25 | | | |
| L x ID (mm) | | 30,000 x 0.25 | | | |
| Flow | | 1 mL/min | | | |

## ***S2.2 Volatile Organic Compounds***

**Table S3.** VOC GC-EI/MS method and validation parameters. Superscripts indicate what compound is used for internal calibration.

| **VOC Compound** | | | | **GC/MS Parameters** | | | | | |
| --- | --- | --- | --- | --- | --- | --- | --- | --- | --- |
| **Analyte** | **VOC Name** | **CAS #s** | **M.W. (g/mol)** | **RTs (min)** | **SIM Target Ion** | **Qual Ions** | **LOD (pg/µL)** | **LOQ (pg/µL)** | **Data Flags** |
| 1 | xylenes (m+p)^a^ | 108-38-3, 106-42-3 | 106.17 | 8.197 | 91 | 105.95, 104.95 | 0.73 | 3.66 | J |
| 2 | styrene^a^ | 100-42-5 | 104.15 | 8.711 | 104 | 103, 78 | 0.65 | 3.24 | J |
| 3 | 2-chlorotoluene^a^ | 95-49-8 | 126.58 | 10.084 | 91 | 125.95, 128 | 0.70 | 3.49 | J |
| 4 | n-propylbenzene^a^ | 103-65-1 | 120.20 | 10.161 | 91 | 120, 65 | 0.69 | 3.47 | J |
| 5 | 4-chlorotoluene^a^ | 106-43-4 | 126.58 | 10.260 | 91 | 125.95, 128 | 0.68 | 3.42 | J |
| 6 | 1,3,5-trimethylbenzene^a^ | 108-67-8 | 120.20 | 10.522 | 105 | 120, 77 | 0.69 | 3.46 | J,B |
| 7 | tert-butylbenzene^a^ | 98-06-6 | 134.22 | 11.013 | 119 | 91, 133.95 | 0.72 | 3.61 |  |
| 8 | 1,2,4-trimethylbenzene^b^ | 95-63-6 | 120.20 | 11.067 | 105 | 120, 77 | 0.70 | 3.50 | B |
| 9 | 1,3 (m)-dichlorobenzene^a^ | 541-73-1 | 147.00 | 11.324 | 146 | 148, 111 | 0.67 | 3.37 |  |
| 10 | sec-butylbenzene^a^ | 135-98-8 | 134.22 | 11.412 | 105 | 133.95, 90.95 | 0.70 | 3.51 |  |
| 11 | 1,4 (p)-dichlorobenzene^a^ | 106-46-7 | 147.00 | 11.534 | 146 | 147.85, 110.9 | 3.39 | 16.97 | B |
| 12 | 1,2,3-trimethylbenzene^a^ | 526-73-8 | 120.20 | 11.649 | 105 | 120, 91 | 0.71 | 3.53 | J,B |
| 13 | p-isopropyltoluene^b^ | 99-87-6 | 134.22 | 11.750 | 119 | 133.95, 91 | 0.70 | 3.50 | J,B |
| 14 | 1,2 (o)-dichlorobenzene^b^ | 95-50-1 | 147.00 | 11.906 | 146 | 147.85, 110.85 | 0.69 | 3.45 |  |
| 15 | n-butylbenzene^b^ | 104-51-8 | 134.22 | 12.400 | 91 | 91.95, 133.95 | 0.72 | 3.61 | J,B |
| 16 | 1,2,4-trichlorobenzene^b^ | 120-82-1 | 181.44 | 14.378 | 182 | 180, 145 | 0.72 | 3.58 |  |
| 17 | n-dodecane^b^ | 112-40-3 | 170.34 | 14.640 | 71 | 85.1, 170.1 | 0.79 | 3.94 | B |
| 18 | 1,2,3-trichlorobenzene^b^ | 87-61-6 | 181.44 | 14.801 | 180 | 181.85, 145 | 0.73 | 3.67 |  |
| 19 | n-tetradecane^b^ | 629-59-4 | 198.39 | 16.745 | 71 | 85.1, 198.1 | 0.82 | 4.08 | B |
| 20 | n-pentadecane^b^ | 629-62-9 | 212.42 | 17.606 | 71 | 85.1, 212.2 | 0.82 | 4.09 | B |
| 21 | n-hexadecane^a^ | 544-76-3 | 226.45 | 18.403 | 71 | 85.1, 226.2 | 0.82 | 4.12 | B |
| **Internal Standards** | | | | | | | | | |
| perylene-d12 (IS) | | 1520-96-3 | 264.38 | 25.748 | 264 | 265 |  |  |  |
| 1,4-dichlorobenzene-d4 (a) | | 3855-82-1 | 151.03 | 11.493 | 152 | 150 |  |  |  |
| naphthalene-d8 (b) | | 1146-65-2 | 136.22 | 14.397 | 136 | 137 |  |  |  |

Limits of detection (LOD) were calculated from 10 over-spiked wristbands (500ppb) extracted, analyzed over 5 days for a total of 22 different injections. LOD calculation RSD * Lowest Cal * Student T for 99% confidence level (2.5176). A data flag of “B” means that background concentrations of the analyte were found in QC samples, and a background correction was applied. A data flag of “J” means that the recovery of the compound from the silicone polymer through extraction and solid-phase extraction is reduced (30-70% recovery).

**Table S4:** VOC GC/MS Operating Parameters

| **Carrier Gas:** Helium (99.99% Pure minimum) | | | | |
| --- | --- | --- | --- | --- |
| **Column Settings & Specifications** | | | **Oven Program** | |
| **Column type** | | J&W DB-5MS, Part # 122-5532, Film 0.25 µm, ID 0.25mm, 30m | **Temp (°C) and Time (min) program** | |
|  |  |  | 35^o^C, 4.00 min hold | |
| **Inlet Parameters** | | | 8^o^C/min to 100^o^C | |
| **Injection Type** | | Splitless | 16^o^C/min to 340^o^C | |
| **MMI Injector Temp** | | 100°C | 340^o^C, 4.00min hold | |
| **Constant Flow Mode** | | 1.3 ml/min | 31.13 min Total Run Time | |
| **Other Settings** | | | | |
| **Heating elements** | | **Value (°C)** | | **Maximum** |
| **Thermal Auxillary** | | 240^o^C | | **-** |
| **MS Source** | | 300^o^C | | 300^o^C |
| **MS Quad** | | 180^o^C | | 200^o^C |
| **SIM Group Parameters** | | | | |
| **Group #** | **Start Time/Dwell** | **Ions** | | |
| 1 | 2.85 min/ 30 | 50.0, 51.0, 57.0, 71.0, 78.0, 82.0, 84.0, 100.0 | | |
| 2 | 5.00 min/ 30 | 65.0, 71.0, 85.0, 91.0, 92.0, 98.0, 100.0, 114.0 | | |
| 3 | 7.20 min/ 25 | 51.0, 78.0, 91.0, 103.0, 104.0, 105.0, 106.0, 112.0, 114.0 | | |
| 4 | 8.75 min/ 15 | 54.0, 62.0, 71.0, 77.0, 82.0, 85.0, 90.0, 92.0, 97.0, 99.0, 105.0, 108.0, 120.0, 128.0 | | |
| 5 | 9.50 min/ 80 | 77.0, 156.0, 158.0 | | |
| 6 | 10.00 min/ 30 | 65.0, 77.0, 91.0, 105.0, 106.0, 120.0, 126.0, 128.0 | | |
| 7 | 10.85 min/ 15 | 71.0, 77.0, 85.0, 91.0, 105.0, 111.0, 119.0, 120.0, 134.0, 142.0, 146.0, 148.0, 150.0, 152.0 | | |
| 8 | 12.20 min/ 80 | 91.0, 92.0, 134.0 | | |
| 9 | 13.00 min/ 15 | 71.0, 85.0, 111.00, 127.0, 128.0, 129.0, 136.0, 137.0, 145.0, 156.0, 170.0, 180.0, 182.0 | | |
| 10 | 15.10 min/ 80 | 115.0, 141.0, 142.0 | | |
| 11 | 16.20 min/ 20 | 71.0, 85.0, 92.0, 102.0, 115.0, 118.0, 130.0, 141.0, 146.0, 156.0, 158.0, 198.0 | | |
| 12 | 17.23 min/ 15 | 71.0, 85.0, 115.0, 122.0, 141.0, 151.0, 152.0, 153.0, 154.0, 156.0, 160.0, 161.0, 180.0, 212.0 | | |
| 13 | 18.00 min/10 | 71.0, 85.0, 99.0, 102.0, 130.0, 154.0, 155.0, 163.0, 165.0, 166.0, 169.0, 174.0, 176.0, 184.0 | | |
| 14 | 19.00 min/ 10 | 71.0, 85.0, 139.0, 151.0, 152.0, 158.0, 160.0, 170.00, 176.0, 178.0, 179.0, 180.0, 184.0 | | |
| 15 | 20.20 min/ 20 | 126.0, 139.0, 151.0, 152.0, 154.0, 168.0, 180.0, 182.0, 189.0, 191.0, 192.0, 196.0 | | |
| 16 | 21.05 min/ 20 | 71.0, 85.0, 126.0, 152.0, 180.0, 188.0, 189.0, 191.0, 192.0, 208.0, 216.0, 282.0 | | |
| 17 | 21.30 min/ 15 | 152.0, 176.0, 180.0, 189.0, 191.0, 200.0, 202.0, 203.0, 204.0, 205.0, 206.0, 208.0, 212.0 | | |
| 18 | 22.25 min/ 10 | 99.0, 112.0, 113.0, 151.0, 152.0, 180.0, 191.0, 193.0, 200.0, 204.0, 213.0, 215.0, 216.0 | | |
| 19 | 23.80 min/ 30 | 112.0, 113.0, 224.0, 226.0, 228.0, 240.0, 241.0 | | |
| 20 | 24.08 min/ 15 | 165.0, 176.0, 202.0, 204.0, 229.0, 230.0, 232.0, 241.0, 242.0, 243.0, 257.0, 258.0, 259.0 | | |
| 21 | 25.00 min/ 35 | 113.0, 226.0, 250.0, 252.0, 253.0, 254.0, 264.0, 265.0 | | |
| 22 | 26.90 min/ 30 | 138.0, 139.0, 274.0, 276.0, 278.0, 279.0, 288.0, 289.0 | | |
| 23 | 29.10 min/ 60 | 150.0, 300.0, 301.0, 302.0 | | |

**Table S5**. VOC background corrections based on lab processing QC sample.

| **Sample Receipt: 4/2/2019** | | **Sample Receipt: 5/15//2019** | |
| --- | --- | --- | --- |
| **Analyte** | **pg/µL** | **Analyte** | **pg/µL** |
| 2-chlorotoluene | 6790 | 1,3,5-trimethylbenzene | 1670 |
| 1,3,5-trimethylbenzene | 2390 | 1,2,4-trimethylbenzene | 1320 |
| 1,2,4-trimethylbenzene | 2120 | 1,4-dichlorobenzene | 47.7 |
| sec-butylbenzene | 141 | 1,2,3-trimethylbenzene | 716 |
| 1,2,3-trimethylbenzene | 895 | p-isopropyltoluene | 34.4 |
| butylbenzene | 113 | butylbenzene | 46 |
| dodecane | 282 | dodecane | 174 |
| tetradecane | 521 | tetradecane | 601 |
| pentadecane | 393 | pentadecane | 503 |
| hexadecane | 1260 | hexadecane | 1530 |

## ***S2.3 Polychlorinated Biphenyls***

**Table S6**. PCB GC-EI/MS method and validation parameters. Superscripts indicate what compound is used for internal calibration.

| **PCB Compound** | | | | | **GC/MS Parameters** | | | |
| --- | --- | --- | --- | --- | --- | --- | --- | --- |
| **Congener** | **Congener Type** | **Chlorine Positions** | **CAS Number** | **Molecular Weight (g/mol)** | **RT (min)** | **SIM Target Ion** | **LOD (pg/µL)** | **LOQ (pg/µL)** |
| PCB 1  ^a^ | Mono- | 2 | 2051-60-7 | 188.6 | 12.482 | 188.0 | 0.05 | 0.25 |
| PCB 10 ^a^ | Di- | 2,6 | 33146-45-1 | 223.1 | 13.948 | 222.0 | 0.07 | 0.34 |
| PCB 8 ^a^ | Di- | 2,4' | 34883-43-7 | 223.1 | 15.016 | 222.0 | 0.07 | 0.34 |
| PCB 50 ^a^ | Tetra- | 2,2',4,6 | 62796-65-0 | 292.0 | 17.264 | 291.9 | 0.09 | 0.43 |
| PCB 28 ^a^ | Tri- | 2,4,4' | 7012-37-5 | 257.5 | 17.369 | 255.9 | 0.08 | 0.39 |
| PCB 21 ^a^ | Tri- | 2,3,4 | 55702-46-0 | 257.5 | 17.552 | 255.9 | 0.08 | 0.40 |
| PCB 52 ^a^ | Tetra- | 2,2',5,5' | 35693-99-3 | 292.0 | 18.31 | 291.9 | 0.05 | 0.23 |
| PCB 49 ^a^ | Tetra- | 2,2',4,5' | 41464-40-8 | 292.0 | 18.415 | 291.9 | 0.08 | 0.40 |
| PCB 44 ^a^ | Tetra- | 2,2',3,5' | 41464-39-5 | 292.0 | 18.694 | 291.9 | 0.08 | 0.42 |
| PCB 37 ^a^ | Tri- | 3,4,4' | 38444-90-5 | 257.5 | 18.835 | 291.9 | 0.07 | 0.35 |
| PCB 104 ^b^ | Penta- | 2,2',4,6,6' | 56558-16-8 | 326.4 | 19.008 | 255.9 | 0.08 | 0.40 |
| PCB 74 ^b^ | Tetra- | 2,4,4',5 | 32690-93-0 | 292.0 | 19.875 | 291.9 | 0.08 | 0.40 |
| PCB 70 ^b^ | Tetra- | 2,3',4',5 | 32598-11-1 | 292.0 | 19.995 | 291.9 | 0.08 | 0.40 |
| PCB 66 ^b^ | Tetra- | 2,3',4,4' | 32598-10-0 | 292.0 | 20.06 | 291.9 | 0.08 | 0.38 |
| PCB 60 ^b^ | Tetra- | 2,3,4,4' | 33025-41-1 | 292.0 | 20.537 | 291.9 | 0.09 | 0.47 |
| PCB 101 ^b^ | Penta- | 2,2',4,5,5' | 37680-73-2 | 326.4 | 20.726 | 325.9 | 0.12 | 0.58 |
| PCB 99 ^b^ | Penta- | 2,2',4,5,5' | 38380-01-7 | 326.4 | 20.862 | 325.9 | 0.09 | 0.43 |
| PCB 145 ^b^ | Hexa- | 2,2',3,4,6,6' | 74472-40-5 | 360.9 | 21.416 | 359.8 | 0.06 | 0.30 |
| PCB 87 ^b^ | Penta- | 2,2',3,4,5' | 38380-02-8 | 326.4 | 21.469 | 325.9 | 0.15 | 0.75 |
| PCB 81 ^b^ | Tetra- | 3,4,4',5 | 70362-50-4 | 292.0 | 21.508 | 291.9 | 0.15 | 0.73 |
| PCB 77 ^b^ | Tetra- | 3,3',4,4' | 32598-13-3 | 292.0 | 21.796 | 291.9 | 0.17 | 0.86 |
| PCB 82 ^b^ | Penta- | 2,2',3,3',4 | 52663-62-4 | 326.4 | 22.024 | 325.9 | 0.08 | 0.38 |
| PCB 123 ^b^ | Penta- | 2',3,4,4',5 | 65510-44-3 | 326.4 | 22.412 | 325.9 | 0.08 | 0.40 |
| PCB 118 ^b^ | Penta- | 2,3',4,4',5 | 31508-00-6 | 326.4 | 22.522 | 325.9 | 0.11 | 0.57 |
| PCB 114 ^b^ | Penta- | 2,3,4,4',5 | 74472-37-0 | 326.4 | 22.799 | 325.9 | 0.06 | 0.31 |
| PCB 153 ^b^ | Hexa- | 2,2',4,4',5,5' | 35065-27-1 | 360.9 | 23.144 | 359.8 | 0.04 | 0.21 |
| PCB 105 ^b^ | Penta- | 2,3,3',4,4' | 32598-14-4 | 326.4 | 23.242 | 325.9 | 0.09 | 0.47 |
| PCB 179 ^b^ | Hepta- | 2,2',3,3',5,6,6' | 52663-64-6 | 395.3 | 23.492 | 393.8 | 0.11 | 0.56 |
| PCB 138+158 ^b^ | Hexa- | 2,3,3',4,4',6 + 2,2',3,4,4',5' | 74472-42-7,  35065-28-2 | 360.9 | 23.938 | 359.8 | 0.09 | 0.43 |
| PCB 126 ^c^ | Penta- | 3,3',4,4',5 | 57465-28-8 | 326.4 | 24.201 | 325.9 | 0.14 | 0.69 |
| PCB 166 ^c^ | Hexa- | 2,3,4,4',5,6 | 41411-63-6 | 360.9 | 24.277 | 359.8 | 0.25 | 1.24 |
| PCB 187 ^b^ | Hepta- | 2,2',3,4',5,5',6 | 52663-68-0 | 395.3 | 24.342 | 393.8 | 0.08 | 0.42 |
| PCB 183 ^b^ | Hepta- | 2,2',3,4,4',5',6' | 52663-69-1 | 395.3 | 24.499 | 393.8 | 0.12 | 0.58 |
| PCB 128 ^b^ | Hexa- | 2,2',3,3',4,4' | 38380-07-3 | 360.9 | 24.656 | 359.8 | 0.12 | 0.59 |
| PCB 167 ^c^ | Hexa- | 2,3',4,4',5,5' | 52663-72-6 | 360.9 | 24.748 | 359.8 | 0.19 | 0.95 |
| PCB 156 ^b^ | Hexa- | 2,3,3',4,4',5 | 38380-08-4 | 360.9 | 25.374 | 359.8 | 0.07 | 0.36 |
| PCB 204 ^d^ | Octa- | 2,2',3,4,4',5,6,6' | 74472-52-9 | 429.8 | 25.509 | 429.8 | 0.10 | 0.51 |
| PCB 157 ^b^ | Hexa- | 2,3,3',4,4',5' | 69782-90-7 | 360.9 | 25.536 | 359.8 | 0.08 | 0.39 |
| PCB 180 ^c^ | Hepta- | 2,2',3,4,4',5,5' | 35065-29-3 | 395.3 | 25.846 | 393.8 | 0.18 | 0.91 |
| PCB 169 ^c^ | Hexa- | 3,3',4,4',5,5' | 32774-16-6 | 360.9 | 26.461 | 359.8 | 0.14 | 0.68 |
| PCB 170 ^c^ | Hepta- | 2,2',3,3',4,4',5 | 35065-30-6 | 395.3 | 26.634 | 393.8 | 0.18 | 0.91 |
| PCB 189 ^d^ | Hepta- | 2,3,3',4,4',5,5' | 39635-31-9 | 395.3 | 27.487 | 393.8 | 0.09 | 0.44 |
| **Internal Standards** | | | | | | | | |
| Perylene-d12 (IS) | | | 1520-96-3 | 264.4 | 30.367 | 264.1 |  | |
| Phenanthrene-d10 SS (a) | | | 1517-22-2 | 188.0 | 16.133 | 188.0 |  |  |
| PCB 100 SS (b) | | | 39485-83-1 | 326.4 | 19.55 | 325.9 |  |  |
| Chrysene-d12 SS (c) | | | 1719-03-5 | 240.0 | 25.329 | 240.1 |  |  |
| PCB 209 SS (d) | | | 2051-24-3 | 498.6 | 30.259 | 497.7 |  |  |

**Table S7:** PCB GC-MS control parameters

| **Carrier Gas:** Helium (99.99%) | | **System Makeup Gas:** Nitrogen | |
| --- | --- | --- | --- |
| **Oven Program** | | **Inlet Parameters** | |
| **Temperature (oC) and Time (min) program** | | **Column Settings & Specifications** | |
| 60^o^C initial, 1.00 min hold; 10^o^C/min to 180^o^C; 6^o^C/min to 310^o^C; 310^o^C, hold for 3 min | | Agilent DB-5MS, 30m, 0.25mm, Film 0.25μm | |
| 37.67 min Total Run Time | | Constant Flow | 1.2 mL/min |
| **Heating Elements** | | **Value (oC)** | **Maximum** |
| Thermal Auxiliary (MSD Transfer Line) | | 280 | n/a |
| MS Source | | 300 | 300 |
| MS Quad | | 180 | 180 |
| **Other Settings** | | | |
| Injection volume (μL) | | 1 | |
| Mode | | Pulsed Splitless | |
| Heater (°C) | | 300 | |
| Total flow (psi) | | 10.42 | |
| Septum purge flow (mL/min) | | 24.2 | |
| Injection pulse pressure (psi) | | 25 until 0.5 min | |
| Purge flow to split vent (mL/min) | | 20 at 0.45 min | |
| Transfer line (°C) | | 280 | |
| Average velocity (cm/s) | | 40.12 | |
| **SIM Group Parameters (Acquisition Mode=SIM/Scan)** | | | |
| **SIM Ion Group #** | **Start Time/Dwell** | **SIM Ions** | |
| Group 1 | 9.00 min/ 10 | 152.0, 188.0, 190.0, 222.0, 224.0, 230.0, 232.0 | |
| Group 2 | 15.20 min/ 10 | 184.1, 186.0, 188.1, 189.1, 219.9, 255.9, 257.9, 290.0, 291.9 | |
| Group 3 | 18.55 min/ 10 | 186.0, 219.9, 255.9, 257.9, 289.9, 291.9, 323.9, 325.9, 327.9 | |
| Group 4 | 19.70 min/ 10 | 208.1, 212.1, 213.1, 219.9, 289.9, 291.9, 323.9, 325.9, 327.9 | |
| Group 5 | 21.20 min/ 10 | 219.9, 253.9, 255.9, 289.9, 291.9, 325.9, 359.8, 361.8 | |
| Group 6 | 21.90 min/ 10 | 253.9, 255.9, 289.9, 323.9, 325.9, 327.9, 359.8, 361.8 | |
| Group 7 | 23.35 min/ 10 | 323.8, 357.8, 359.8, 361.8, 393.8, 395.8 | |
| Group 8 | 24.10 min/ 8 | 255.9, 289.9, 323.8, 325.9, 359.8, 361.8, 393.8, 395.8 | |
| Group 9 | 25.00 min/ 8 | 236.1, 240.1, 241.1, 289.9, 357.8, 359.8, 361.8, 427.8, 429.8 | |
| Group 10 | 25.70 min/ 10 | 289.9, 323.8, 359.8, 361.8, 393.8, 395.8 | |
| Group 11 | 27.80 min/ 10 | 260.1, 264.1, 265.2, 495.7, 497.7, 49 | |

# **S3. Additional notes on quantitation**

**Table S8.** Data quality objectives, acceptance criteria, and corrective actions for all methods

| **QC Sample** | **Purpose** | **Frequency** | **Acceptance Criteria** | **Corrective Action** |
| --- | --- | --- | --- | --- |
| **Initial Calibration** | Accuracy | Prior to project analyses and if post-maintenance calibration check fails | R^2^ of ≥0.99 if linear, ≥0.995 if quadratic | Perform instrument maintenance, reanalyze |
| **Instrument Detection Limits** | Detection Limit | Prior to project analyses | Signal to noise (peak to peak) ratio is above 3 | Perform instrument maintenance, reanalyze |
| **Continuing Calibration Verification** | Accuracy despite matrix or instrument variability | One per 10-15 samples or as necessary | ±30% for at least 80% of target analytes | Perform instrument maintenance, reanalyze samples |
| **Instrument Blank** | Detection limit, contamination | Beginning of each batch | Below analyte instrument detection limit | Perform instrument maintenance, reanalyze, flag data |
| **Sample Spike or Duplicate Sample** | Preparation and handling bias | One per 25 field samples | Analyte percent recovery 50% to 150% | Flag data as appropriate |
| **Internal Standard Area Counts** | Analytical bias | Each sample and standard | Percent detected 50% to 100% | Reanalyze if a standard, perform instrument maintenance, and/or flag data |
| **Surrogate Recovery** | Analytical bias | Each sample | Percent recovered 50% to 150% | Reanalyze if a blank or standard, flag sample data |

**Table S9.** VOC estimated time to equilibrium – Reference: O’Connell et al 2022 (3)

| **VOC Analytes** | **Boiling Point (BP) (Celsius)** | | **log ke based on BP regression (OPERA)**  **-0.009*BP + 1.55** | | **K_e_ (day^-1^)** | | **Estimated time to equilibrium (days)**  **(LN(2)/ke)*4** | | **Approximate Exposure Window (last X days of sampling period rounded to 0.5 days)** |
| --- | --- | --- | --- | --- | --- | --- | --- | --- | --- |
|  | **lower range** | **upper range** | **Lower** | **Upper** | **Upper** | **Lower** | **Lower** | **Upper** |  |
| toluene | 111 | -- | 0.551 | -- | 3.56 | -- | 0.78 | -- | <1 |
| xylenes (m p) | 141 | -- | 0.281 | -- | 1.91 | -- | 1.45 | -- | 1.5 |
| 1,2,4-trimethylbenzene | 169 | 170 | 0.029 | 0.020 | 1.07 | 1.05 | 2.59 | 2.65 | 2.5 |
| 1,3-dichlorobenzene | 173 | 175 | -0.007 | -0.025 | 0.98 | 0.94 | 2.82 | 2.94 | 2.0 |
| 1,3,5-trimethylbenzene | 165 | 170 | 0.065 | 0.020 | 1.16 | 1.05 | 2.39 | 2.65 | 2.0 |
| 1,4-dichlorobenzene | 174 | 175 | -0.016 | -0.025 | 0.96 | 0.94 | 2.88 | 2.94 | 3.0 |
| 1,2,3-trimethylbenzene | 170 | 176 | 0.020 | -0.034 | 1.05 | 0.92 | 2.65 | 3.00 | 3.0 |
| 1,2-dichlorobenzene | 175 | 180 | -0.025 | -0.070 | 0.94 | 0.85 | 2.94 | 3.26 | 3.0 |
| sec-butylbenzene | 174 | 178 | -0.016 | -0.052 | 0.96 | 0.89 | 2.88 | 3.13 | 3.0 |
| tert-butylbenzene | 170 | -- | 0.020 | -- | 1.05 | -- | 2.65 | -- | 2.5 |
| p-isopropyltoluene | 176 | 178 | -0.034 | -0.052 | 0.92 | 0.89 | 3.00 | 3.13 | 3.0 |
| butylbenzene | 183 | 190 | -0.097 | -0.160 | 0.80 | 0.69 | 3.47 | 4.01 | 3.5 |
| dodecane | 205 | 216 | -0.295 | -0.394 | 0.51 | 0.40 | 5.47 | 6.87 | 6.0 |
| pentadecane | 260 | 270 | -0.790 | -0.880 | 0.16 | 0.13 | 17.1 | 21.0 | 19 |
| octane | 125 | 126 | 0.425 | 0.416 | 2.66 | 2.61 | 1.04 | 1.06 | 1.0 |
| 2-chlorotoluene | 159 | -- | 0.119 | -- | 1.32 | -- | 2.11 | -- | 2.0 |
| n-propylbenzene | 170 | -- | 0.020 | -- | 1.05 | -- | 2.65 | -- | 2.0 |
| 4-chlorotoluene | 162 | -- | 0.092 | -- | 1.24 | -- | 2.24 | -- | 2.0 |
| hexadecane | 277 | 287 | -0.943 | -1.033 | 0.11 | 0.09 | 24.3 | 29.9 | 27 |

*Approximate exposure window represents the average of the lower and upper estimates, rounded to closest 0.5 day

# **S4. Data tables**

**Table S10.** Summary of detections for nine potential “occupational exposures” by firefighter rank, years in the fire service, and number of fire attacks. Firefighter counts do not add up to the total participant count in some cases due to missing responses.

| **Rank** | **Station** | **Duty Status** | **PBDE 47** | | **PBDE 99** | | **PBDE 100** | | **PBDE 28+33** | | **PBDE 49** | | **sec-butylbenzene** | |
| --- | --- | --- | --- | --- | --- | --- | --- | --- | --- | --- | --- | --- | --- | --- |
| **Operational Firefighter** | High Call n=16 | Off | 12 | 75% | 8 | 50% | 2 | 13% | 0 | 0% | 0 | 0% | 0 | 0% |
| 34 firefighters; 68 samples |  | On | 16 | 100% | 14 | 88% | 5 | 31% | 0 | 0% | 0 | 0% | 2 | 13% |
|  | Low Call n=18 | Off | 17 | 94% | 11 | 61% | 7 | 39% | 0 | 0% | 0 | 0% | 3 | 17% |
|  |  | On | 18 | 100% | 18 | 100% | 16 | 89% | 9 | 50% | 6 | 33% | 7 | 39% |
|  | **Total** | | 63 | 93% | 51 | 75% | 30 | 44% | 9 | 13% | 6 | 9% | 12 | 18% |
| **Captain** | High Call n=7 | Off | 5 | 71% | 4 | 57% | 3 | 43% | 0 | 0% | 0 | 0% | 1 | 14% |
| 13 firefighters; 26 samples |  | On | 7 | 100% | 5 | 71% | 4 | 57% | 0 | 0% | 0 | 0% | 1 | 14% |
|  | Low Call n=6 | Off | 6 | 100% | 4 | 67% | 3 | 50% | 0 | 0% | 0 | 0% | 2 | 33% |
|  |  | On | 6 | 100% | 6 | 100% | 4 | 67% | 3 | 50% | 2 | 33% | 3 | 50% |
|  |  | **Total** | 24 | 92% | 19 | 73% | 14 | 54% | 3 | 12% | 2 | 8% | 7 | 27% |
| **Chief** | High Call n=4 | Off | 4 | 100% | 2 | 50% | 1 | 25% | 1 | 25% | 1 | 25% | 0 | 0% |
| 7 firefighters; 14 samples |  | On | 4 | 100% | 4 | 100% | 1 | 25% | 1 | 25% | 1 | 25% | 0 | 0% |
|  | Low Call n=3 | Off | 1 | 33% | 1 | 33% | 0 | 0% | 0 | 0% | 0 | 0% | 1 | 33% |
|  |  | On | 2 | 67% | 1 | 33% | 0 | 0% | 0 | 0% | 0 | 0% | 1 | 33% |
|  |  | **Total** | 11 | 79% | 8 | 57% | 2 | 14% | 2 | 14% | 2 | 14% | 2 | 14% |
|  |  |  |  |  |  |  |  |  |  |  |  |  |  |  |
| **Years in the Fire Service (binned)** | **Station** | **Duty Status** | **PBDE 47** | | **PBDE 99** | | **PBDE 100** | | **PBDE 28+33** | | **PBDE 49** | | **sec-butylbenzene** | |
| **1 -9 years** | High Call n=10 | Off | 7 | 70% | 6 | 60% | 2 | 20% | 0 | 0% | 0 | 0% | 0 | 0% |
| 17 firefighters; 34 samples |  | On | 10 | 100% | 8 | 80% | 2 | 20% | 0 | 0% | 0 | 0% | 2 | 20% |
|  | Low Call n=7 | Off | 6 | 86% | 5 | 71% | 4 | 57% | 0 | 0% | 0 | 0% | 0 | 0% |
|  |  | On | 7 | 100% | 7 | 100% | 6 | 86% | 4 | 57% | 4 | 57% | 2 | 29% |
|  | **Total** | | 30 | 88% | 26 | 76% | 14 | 41% | 4 | 12% | 4 | 12% | 4 | 12% |
| **10-19 years** | High Call n=7 | Off | 7 | 100% | 3 | 43% | 1 | 14% | 0 | 0% | 0 | 0% | 0 | 0% |
| 21 firefighters; 42 samples |  | On | 7 | 100% | 6 | 86% | 2 | 29% | 0 | 0% | 0 | 0% | 0 | 0% |
|  | Low Call n=14 | Off | 12 | 86% | 7 | 50% | 4 | 29% | 0 | 0% | 0 | 0% | 5 | 36% |
|  |  | On | 13 | 93% | 12 | 86% | 9 | 64% | 6 | 43% | 3 | 21% | 6 | 43% |
|  |  | **Total** | 39 | 93% | 28 | 67% | 16 | 38% | 6 | 14% | 3 | 7% | 11 | 26% |
| **20-29 years** | High Call n=5 | Off | 3 | 60% | 3 | 60% | 2 | 40% | 1 | 20% | 1 | 20% | 1 | 20% |
| 11 firefighters; 22 samples |  | On | 5 | 100% | 4 | 80% | 3 | 60% | 1 | 20% | 1 | 20% | 1 | 20% |
|  | Low Call n=6 | Off | 6 | 100% | 4 | 67% | 2 | 33% | 0 | 0% | 0 | 0% | 1 | 17% |
|  |  | On | 6 | 100% | 6 | 100% | 5 | 83% | 2 | 33% | 1 | 17% | 3 | 50% |
|  |  | **Total** | 20 | 91% | 17 | 77% | 12 | 55% | 4 | 18% | 3 | 14% | 6 | 27% |
| **> 30 years** | High Call n=2 | Off | 2 | 100% | 1 | 50% | 0 | 0% | 0 | 0% | 0 | 0% | 0 | 0% |
| 2 firefighters; 4 samples |  | On | 2 | 100% | 2 | 100% | 0 | 0% | 0 | 0% | 0 | 0% | 0 | 0% |
|  | Low Call n=0 | Off |  |  |  |  |  |  |  |  |  |  |  |  |
|  |  | On |  |  |  |  |  |  |  |  |  |  |  |  |
|  |  | **Total** | 4 | 100% | 3 | 75% | 0 | 0% | 0 | 0% | 0 | 0% | 0 | 0% |
|  |  |  |  |  |  |  |  |  |  |  |  |  |  |  |
| **Fire attacks participated in (binned)** | **Station** | **Duty Status** | **PBDE 47** | | **PBDE 99** | | **PBDE 100** | | **PBDE 28+33** | | **PBDE 49** | | **sec-butylbenzene** | |
| **None** | High Call n=5 | Off | 5 | 100% | 2 | 40% | 1 | 20% | 0 | 0% | 0 | 0% | 0 | 0% |
| 5 firefighters; 10 samples |  | On | 5 | 100% | 4 | 80% | 1 | 20% | 0 | 0% | 0 | 0% | 0 | 0% |
|  | Low Call n=0 | Off |  |  |  |  |  |  |  |  |  |  |  |  |
|  |  | On |  |  |  |  |  |  |  |  |  |  |  |  |
|  | **Total** | | 10 | 100% | 6 | 60% | 2 | 20% | 0 | 0% | 0 | 0% | 0 | 0% |
| **1-9 Fires** | High Call n=3 | Off | 2 | 67% | 2 | 67% | 1 | 33% | 1 | 33% | 1 | 33% | 0 | 0% |
| 24 firefighters; 48 samples |  | On | 3 | 100% | 2 | 67% | 1 | 33% | 1 | 33% | 1 | 33% | 0 | 0% |
|  | Low Call n=21 | Off | 19 | 90% | 12 | 57% | 7 | 33% | 0 | 0% | 0 | 0% | 3 | 14% |
|  |  | On | 20 | 95% | 20 | 95% | 16 | 76% | 8 | 38% | 5 | 24% | 7 | 33% |
|  |  | **Total** | 44 | 92% | 36 | 75% | 25 | 52% | 10 | 21% | 7 | 15% | 10 | 21% |
| **10-19 Fires** | High Call n=12 | Off | 7 | 58% | 4 | 33% | 2 | 17% | 0 | 0% | 0 | 0% | 1 | 8% |
| 18 firefighters; 36 samples |  | On | 12 | 100% | 10 | 83% | 4 | 33% | 0 | 0% | 0 | 0% | 2 | 17% |
|  | Low Call n=6 | Off | 5 | 83% | 4 | 67% | 3 | 50% | 0 | 0% | 0 | 0% | 3 | 50% |
|  |  | On | 6 | 100% | 5 | 83% | 4 | 67% | 4 | 67% | 3 | 50% | 4 | 67% |
|  |  | **Total** | 30 | 83% | 23 | 64% | 13 | 36% | 4 | 11% | 3 | 8% | 10 | 28% |
| **20-29 fires** | High Call n=7 | Off | 7 | 100% | 6 | 86% | 2 | 29% | 0 | 0% | 0 | 0% | 0 | 0% |
| 7 firefighters; 14 samples |  | On | 7 | 100% | 7 | 100% | 4 | 57% | 0 | 0% | 0 | 0% | 1 | 14% |
|  | Low Call n=0 | Off |  |  |  |  |  |  |  |  |  |  |  |  |
|  |  | On |  |  |  |  |  |  |  |  |  |  |  |  |
|  |  | **Total** | 14 | 100% | 13 | 93% | 6 | 43% | 0 | 0% | 0 | 0% | 1 | 7% |
|  |  |  |  |  |  |  |  |  |  |  |  |  |  |  |

**Table S11.** Summary of Log_2_FC distributions for nine potential “occupational exposures” by firefighter rank, years of fire service, and number of fire attacks.

| **Condition** | | **Rank** | | | | | | **Years in the Fire Service *(binned)*** | | | | | | | | **Fire attacks participated in *(binned)*** | | | | | | | |
| --- | --- | --- | --- | --- | --- | --- | --- | --- | --- | --- | --- | --- | --- | --- | --- | --- | --- | --- | --- | --- | --- | --- | --- |
|  |  | **Operational Firefighter** | | **Captain** | | **Chief** | | **1 -9 years** | | **10-19 years** | | **20-29 years** | | **> 30 years** | | **None** | | **1-9 Fires** | | **10-19 Fires** | | **20-29 fires** | |
|  |  | *34 firefighters; 68 samples* | | *13 firefighters; 26 samples* | | *7 firefighters; 14 samples* | | *17 firefighters; 34 samples* | | *21 firefighters; 42 samples* | | *11 firefighters; 22 samples* | | *2 firefighters; 4 samples* | | *5 firefighters; 10 samples* | | *24 firefighters; 48 samples* | | *18 firefighters; 36 samples* | | *7 firefighters; 14 samples* | |
| **Station** | | High Call n=16 | Low Call n=18 | High Call n=7 | Low Call n=6 | High Call n=4 | Low Call n=3 | High Call n=10 | Low Call n=7 | High Call n=7 | Low Call n=14 | High Call n=5 | Low Call n=6 | High Call n=2 | Low Call n=0 | High Call n=5 | Low Call n=0 | High Call n=3 | Low Call n=21 | High Call n=12 | Low Call n=6 | High Call n=7 | Low Call n=0 |
| **PBDE 47** | **min** | -0.77 | -0.58 | -0.77 | 0.47 | -0.33 | -0.63 | -1.67 | 1.58 | -0.96 | -0.58 | -0.33 | -0.63 | 0.00 |  | -0.01 |  | -0.33 | -0.63 | -0.96 | 0.47 | -1.67 |  |
|  | **Q1** | -0.16 | 1.41 | 0.11 | 0.81 | -0.09 | -0.32 | 0.08 | 1.74 | -0.44 | 0.95 | 0.10 | 0.50 | 0.04 |  | 0.00 |  | -0.32 | 0.98 | 0.23 | 0.78 | 0.01 |  |
|  | **median** | 1.32 | 2.06 | 0.34 | 1.18 | -0.01 | 0.00 | 1.42 | 4.06 | -0.01 | 1.44 | 1.02 | 0.96 | 0.07 |  | 0.10 |  | -0.32 | 1.64 | 3.01 | 2.17 | 0.84 |  |
|  | **Q3** | 4.63 | 3.34 | 2.55 | 1.45 | 0.04 | 2.47 | 3.93 | 4.56 | 0.45 | 2.35 | 4.09 | 2.34 | 0.11 |  | 0.11 |  | 2.47 | 2.67 | 4.79 | 3.29 | 1.14 |  |
|  | **max** | 7.56 | 5.56 | 6.37 | 2.92 | 0.14 | 4.94 | 5.27 | 5.56 | 0.84 | 4.94 | 6.37 | 3.12 | 0.14 |  | 0.14 |  | 5.27 | 5.56 | 7.56 | 4.94 | 1.47 |  |
| **PBDE 99** | **min** | -4.14 | 0.01 | -1.17 | 0.53 | -0.68 | -0.45 | -4.14 | 1.80 | -1.51 | 0.00 | -0.68 | -0.45 | 0.42 |  | 0.00 |  | -4.14 | -0.45 | -1.51 | 0.00 | -1.17 |  |
|  | **Q1** | 0.04 | 1.61 | 0.00 | 0.72 | 0.14 | -0.22 | -0.13 | 1.88 | -0.59 | 0.68 | 0.00 | 0.14 | 1.42 |  | 0.33 |  | -2.41 | 1.00 | 0.00 | 0.14 | 0.42 |  |
|  | **median** | 1.51 | 3.69 | 0.32 | 1.93 | 2.41 | 0.00 | 1.34 | 3.66 | 1.02 | 2.21 | 0.33 | 1.03 | 2.41 |  | 0.42 |  | -0.68 | 1.92 | 2.96 | 1.70 | 1.02 |  |
|  | **Q3** | 4.94 | 6.41 | 0.67 | 3.89 | 4.45 | 0.00 | 3.87 | 4.02 | 4.76 | 5.52 | 1.01 | 5.11 | 3.41 |  | 4.41 |  | 2.39 | 6.31 | 5.05 | 3.67 | 1.51 |  |
|  | **max** | 7.16 | 7.62 | 5.33 | 5.95 | 4.58 | 0.00 | 5.45 | 6.94 | 4.96 | 7.62 | 5.33 | 7.40 | 4.41 |  | 4.58 |  | 5.45 | 7.62 | 7.16 | 4.23 | 4.93 |  |
| **PBDE 100** | **min** | -0.28 | -0.45 | -0.28 | 0.00 | -0.54 | 0.00 | -5.77 | 0.00 | 0.00 | -0.45 | -0.54 | 0.00 | 0.00 |  | -0.28 |  | -0.54 | -0.45 | -5.77 | 0.00 | -0.33 |  |
|  | **Q1** | 0.00 | 1.60 | 0.00 | 0.12 | -0.13 | 0.00 | 0.00 | 1.73 | 0.00 | 0.00 | -0.28 | 0.18 | 0.00 |  | 0.00 |  | -0.27 | 0.00 | 0.00 | 0.02 | 0.00 |  |
|  | **median** | 0.00 | 3.97 | 0.00 | 0.59 | 0.00 | 0.00 | 0.00 | 2.59 | 0.00 | 1.13 | 0.00 | 2.31 | 0.00 |  | 0.00 |  | 0.00 | 2.59 | 0.00 | 0.28 | 0.00 |  |
|  | **Q3** | 0.95 | 5.24 | 0.08 | 1.95 | 0.00 | 0.00 | 0.00 | 4.32 | 0.00 | 4.53 | 0.00 | 5.07 | 0.00 |  | 0.00 |  | 0.00 | 4.83 | 1.07 | 1.90 | 1.99 |  |
|  | **max** | 4.90 | 7.59 | 3.97 | 5.20 | 0.00 | 0.00 | 4.37 | 6.88 | 4.28 | 7.59 | 3.97 | 6.26 | 0.00 |  | 0.00 |  | 0.00 | 6.88 | 4.90 | 7.59 | 4.37 |  |
| **PBDE 28+33** | **min** | 0.00 | 0.00 | 0.00 | 0.00 | -0.31 | 0.00 | -0.13 | 0.00 | 0.00 | 0.00 | -0.75 | -0.15 | 0.00 |  | 0.00 |  | -0.31 | 0.00 | 0.00 | 0.00 | 0.00 |  |
|  | **Q1** | 0.00 | 0.00 | 0.00 | 0.00 | -0.08 | 0.00 | 0.00 | 0.00 | 0.00 | 0.00 | 0.00 | 0.00 | 0.00 |  | 0.00 |  | -0.15 | 0.00 | 0.00 | 0.75 | 0.00 |  |
|  | **median** | 0.00 | 1.68 | 0.00 | 1.49 | 0.00 | 0.00 | 0.00 | 4.33 | 0.00 | 0.00 | 0.00 | 0.00 | 0.00 |  | 0.00 |  | 0.00 | 0.00 | 0.00 | 3.88 | 0.00 |  |
|  | **Q3** | 0.00 | 4.47 | 0.00 | 3.47 | 0.00 | 0.00 | 0.00 | 4.75 | 0.00 | 3.84 | 0.00 | 2.24 | 0.00 |  | 0.00 |  | 0.00 | 3.91 | 0.00 | 5.22 | 0.00 |  |
|  | **max** | 0.00 | 6.19 | 0.00 | 5.58 | 0.00 | 0.00 | 0.00 | 6.19 | 0.00 | 5.58 | 0.00 | 4.77 | 0.00 |  | 0.00 |  | 0.00 | 6.19 | 0.00 | 5.58 | 0.00 |  |
| **PBDE 49** | **min** | 0.00 | 0.00 | 0.00 | 0.00 | -0.36 | 0.00 | 0.00 | 0.00 | 0.00 | 0.00 | -0.36 | 0.00 | 0.00 |  | 0.00 |  | -0.36 | 0.00 | 0.00 | 0.00 | 0.00 |  |
|  | **Q1** | 0.00 | 0.00 | 0.00 | 0.00 | -0.09 | 0.00 | 0.00 | 0.00 | 0.00 | 0.00 | 0.00 | 0.00 | 0.00 |  | 0.00 |  | -0.18 | 0.00 | 0.00 | 0.00 | 0.00 |  |
|  | **median** | 0.00 | 0.00 | 0.00 | 0.00 | 0.00 | 0.00 | 0.00 | 4.30 | 0.00 | 0.00 | 0.00 | 0.00 | 0.00 |  | 0.00 |  | 0.00 | 0.00 | 0.00 | 1.09 | 0.00 |  |
|  | **Q3** | 0.00 | 4.36 | 0.00 | 1.64 | 0.00 | 0.00 | 0.00 | 4.54 | 0.00 | 0.00 | 0.00 | 0.00 | 0.00 |  | 0.00 |  | 0.00 | 0.00 | 0.00 | 3.96 | 0.00 |  |
|  | **max** | 0.00 | 5.40 | 0.00 | 5.61 | 0.00 | 0.00 | 0.00 | 5.40 | 0.00 | 5.61 | 0.00 | 2.18 | 0.00 |  | 0.00 |  | 0.00 | 5.40 | 0.00 | 5.61 | 0.00 |  |
| **sec-butylbenzene** | **min** | 0.00 | -7.30 | 0.00 | 0.00 | 0.00 | 0.00 | 0.00 | 0.00 | 0.00 | -7.30 | 0.00 | -0.35 | 0.00 |  | 0.00 |  | 0.00 | -0.35 | 0.00 | -7.30 | 0.00 |  |
|  | **Q1** | 0.00 | 0.00 | 0.00 | 0.00 | 0.00 | 0.00 | 0.00 | 0.00 | 0.00 | 0.00 | 0.00 | 0.00 | 0.00 |  | 0.00 |  | 0.00 | 0.00 | 0.00 | 0.05 | 0.00 |  |
|  | **median** | 0.00 | 0.00 | 0.00 | 0.09 | 0.00 | 0.00 | 0.00 | 0.00 | 0.00 | 0.00 | 0.00 | 0.00 | 0.00 |  | 0.00 |  | 0.00 | 0.00 | 0.00 | 0.47 | 0.00 |  |
|  | **Q3** | 0.00 | 5.48 | 0.00 | 0.18 | 0.00 | 0.38 | 0.00 | 3.71 | 0.00 | 0.18 | 0.00 | 5.48 | 0.00 |  | 0.00 |  | 0.00 | 0.00 | 0.00 | 5.58 | 0.00 |  |
|  | **max** | 8.21 | 8.15 | 0.03 | 7.19 | 0.00 | 0.75 | 8.21 | 7.47 | 0.00 | 8.07 | 0.03 | 8.15 | 0.00 |  | 0.00 |  | 0.00 | 8.07 | 8.21 | 8.15 | 7.38 |  |

**Table S12.** Percentile thresholds for classifying chemical exposure as “very low,”, “low,” “medium,” or “high” ordered from low to high proportion of samples below the limit of detection (BLOD).

| **Analyte** | **Proportion BLOD** | **Percentile thresholds between four exposure levels: "very low", "low", "medium", "high"** | | |
| --- | --- | --- | --- | --- |
|  |  | **Percentile 1** | **Percentile 2** | **Percentile 3** |
| 1-methylnaphthalene | 0.01 | 0.25 | 0.50 | 0.75 |
| 2-methylnaphthalene | 0.01 | 0.25 | 0.50 | 0.75 |
| 2-methylphenanthrene | 0.01 | 0.25 | 0.50 | 0.75 |
| dibenzothiophene | 0.01 | 0.25 | 0.50 | 0.75 |
| naphthalene | 0.01 | 0.25 | 0.50 | 0.75 |
| pyrene | 0.01 | 0.25 | 0.50 | 0.75 |
| retene | 0.01 | 0.25 | 0.50 | 0.75 |
| 2-methylanthracene | 0.02 | 0.25 | 0.50 | 0.75 |
| fluoranthene | 0.02 | 0.25 | 0.50 | 0.75 |
| 2-ethylnaphthalene | 0.04 | 0.25 | 0.50 | 0.75 |
| fluorene | 0.04 | 0.25 | 0.50 | 0.75 |
| TPHP | 0.05 | 0.25 | 0.50 | 0.75 |
| TDCIPP | 0.06 | 0.25 | 0.50 | 0.75 |
| PBDE 47 | 0.09 | 0.25 | 0.50 | 0.75 |
| n-Tetradecane | 0.19 | 0.25 | 0.50 | 0.75 |
| 1,2-dimethylnaphthalene | 0.19 | 0.25 | 0.50 | 0.75 |
| 1,4-dimethylnaphthalene | 0.19 | 0.25 | 0.50 | 0.75 |
| 1,5-dimethylnaphthalene | 0.21 | 0.25 | 0.50 | 0.75 |
| 1,2,3-Trimethylbenzene | 0.26 | 0.26 | 0.51 | 0.75 |
| n-Pentadecane | 0.26 | 0.26 | 0.51 | 0.75 |
| 1,3,5-Trimethylbenzene | 0.27 | 0.27 | 0.51 | 0.76 |
| PBDE 99 | 0.28 | 0.28 | 0.52 | 0.76 |
| 1-methylpyrene | 0.29 | 0.29 | 0.52 | 0.76 |
| n-Dodecane | 0.29 | 0.29 | 0.52 | 0.76 |
| 3,6-dimethylphenanthrene | 0.33 | 0.33 | 0.56 | 0.78 |
| benzo[b]fluoranthene | 0.36 | 0.36 | 0.57 | 0.79 |
| anthracene | 0.50 | 0.50 | 0.67 | 0.83 |

# **S5. Supplementary figures**

**B**

**A**

| **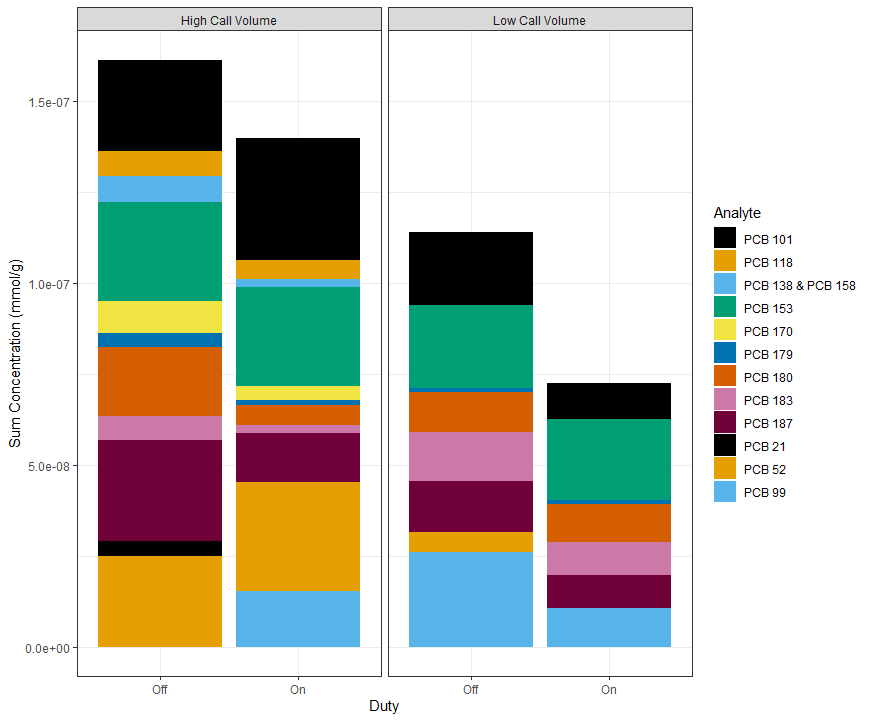** | **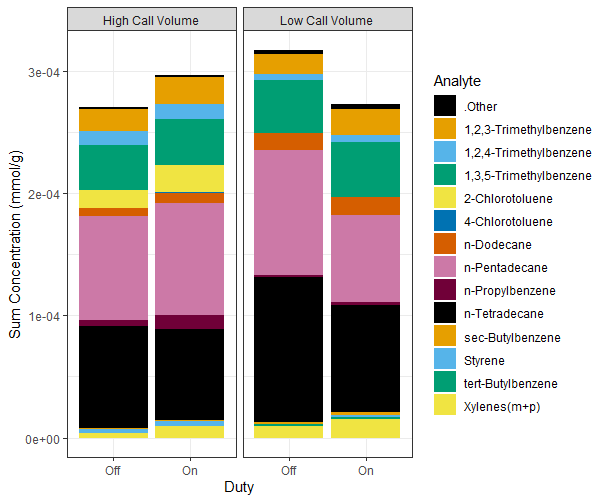** |
| --- | --- |
| **Fig. S1 A** Sum concentrations (mmol/g of silicone) of PCB congeners by station and duty status and **B** Sum concentrations (mmol/g of silicone) of VOCs by station and duty status. For the VOCs, eight analytes with the lowest concentrations across all categories were grouped into an “other” category, shown in black at the top of the bars. | |

**
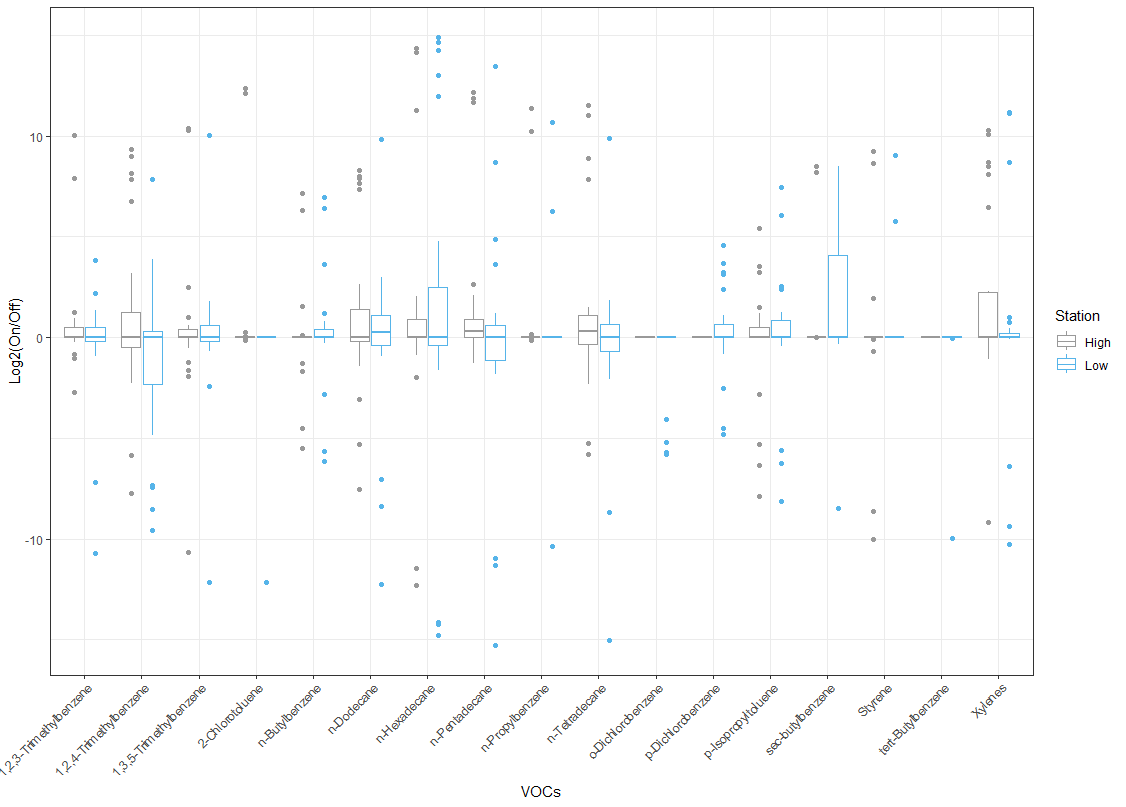
**

**Fig. S2** Log2(fold-change) distributions of detected VOCs between on and off-duty dog tags. Boxplots represent the median, 1^st^ and 3^rd^ quartile, with whiskers to represent the data range, and outliers represented with individual points. Boxplots are colored by station: “High” (grey) corresponds to the high call volume station, and “Low” (blue) corresponds to the low call volume station.


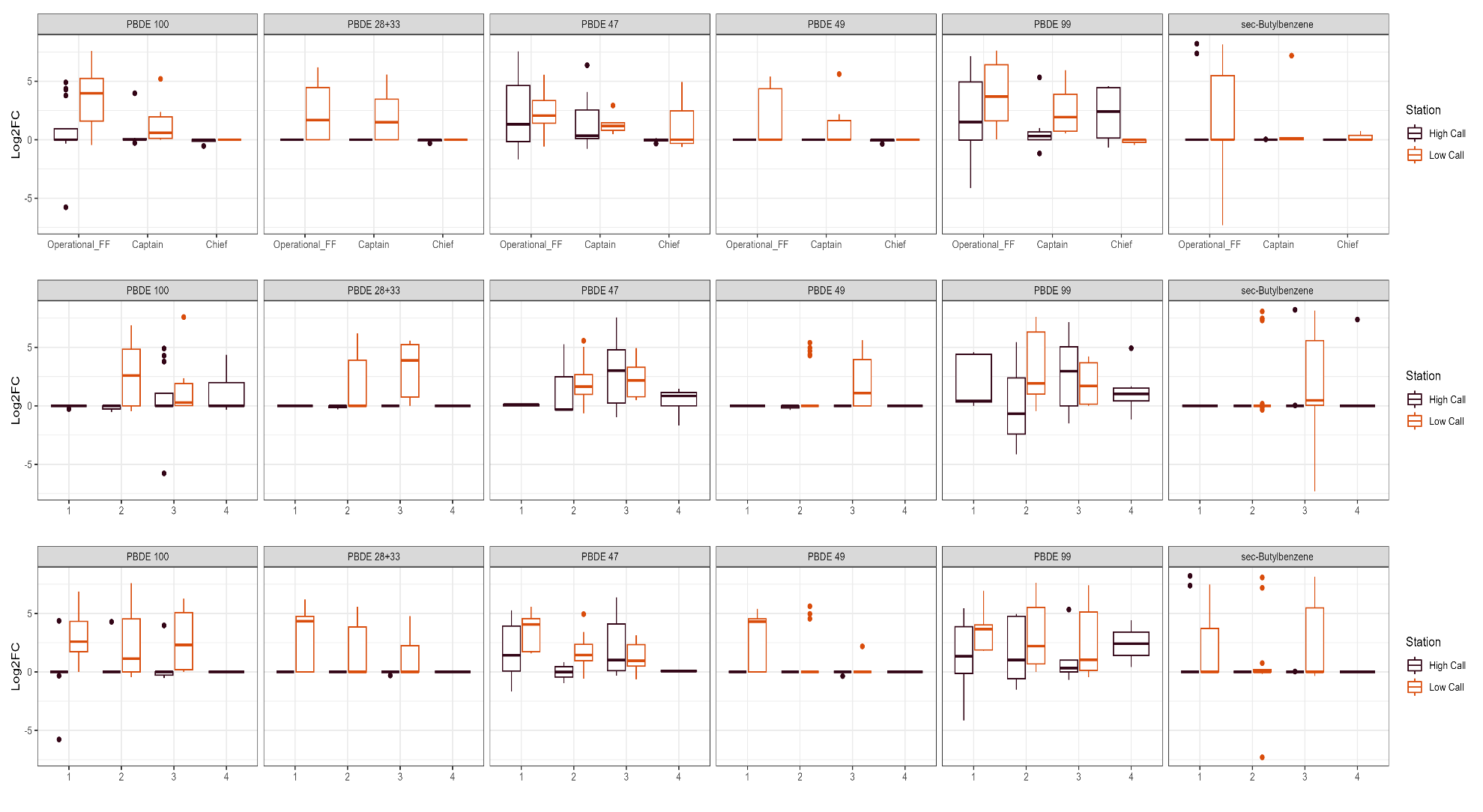


**_B_**

**_C_**

**_A_**

**Fig. S3** Boxplots of Log2FC between on- and off-duty tags in relation to categorical variables **A** rank, **B** number of fires responded to (binned), and **C** years in the fire service (binned), stratified by station. Chemicals identified as potential occupational exposures (excluding PAHs) are included. Categories are defined and described numerically in **Table S10**.


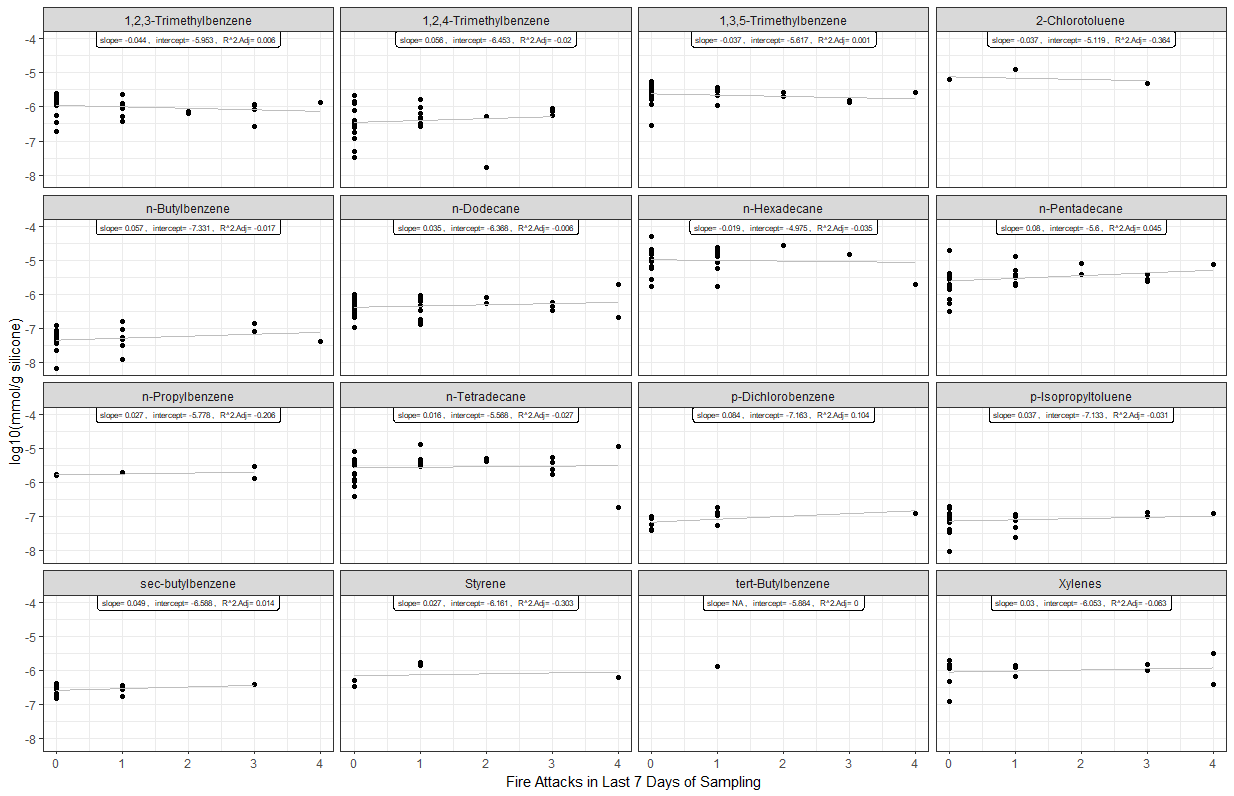


Slope = -0.044, intercept = -5.953, R^2 Adj = 0.006

Slope = 0.056, intercept = -6.453, R^2 Adj = -0.02

Slope = 0.057, intercept = -7.331, R^2 Adj = -0.017

Slope = 0.035, intercept = -6.366, R^2 Adj = -0.006

Slope = -0.037, intercept = -5.617, R^2 Adj = 0.001

Slope = -0.037, intercept = -5.119, R^2 Adj = -0.364

Slope = -0.019, intercept = -4.975, R^2 Adj = -0.035

Slope = 0.08, intercept = -5.6, R^2 Adj = 0.045

Slope = 0.027, intercept = -5.778, R^2 Adj = -0.206

Slope = 0.016, intercept = -5.568, R^2 Adj = -0.027

Slope = 0.064, intercept = -7.163, R^2 Adj = 0.104

Slope = 0.037, intercept = -7.133, R^2 Adj = -0.031

Slope = 0.049, intercept = -6.586, R^2 Adj = 0.014

Slope = 0.027, intercept = -6.161, R^2 Adj = -0.303

Slope =NA, intercept = -5.884, R^2 Adj = -0

Slope = 0.03, intercept = -6.053, R^2 Adj = -0.063

**Fig. S4** Fire attacks participated in over the last seven days of sampling versus concentration of sixteen VOCs in on-duty tags of firefighters from both stations (Log_10_(mmol/g silicone)).

| **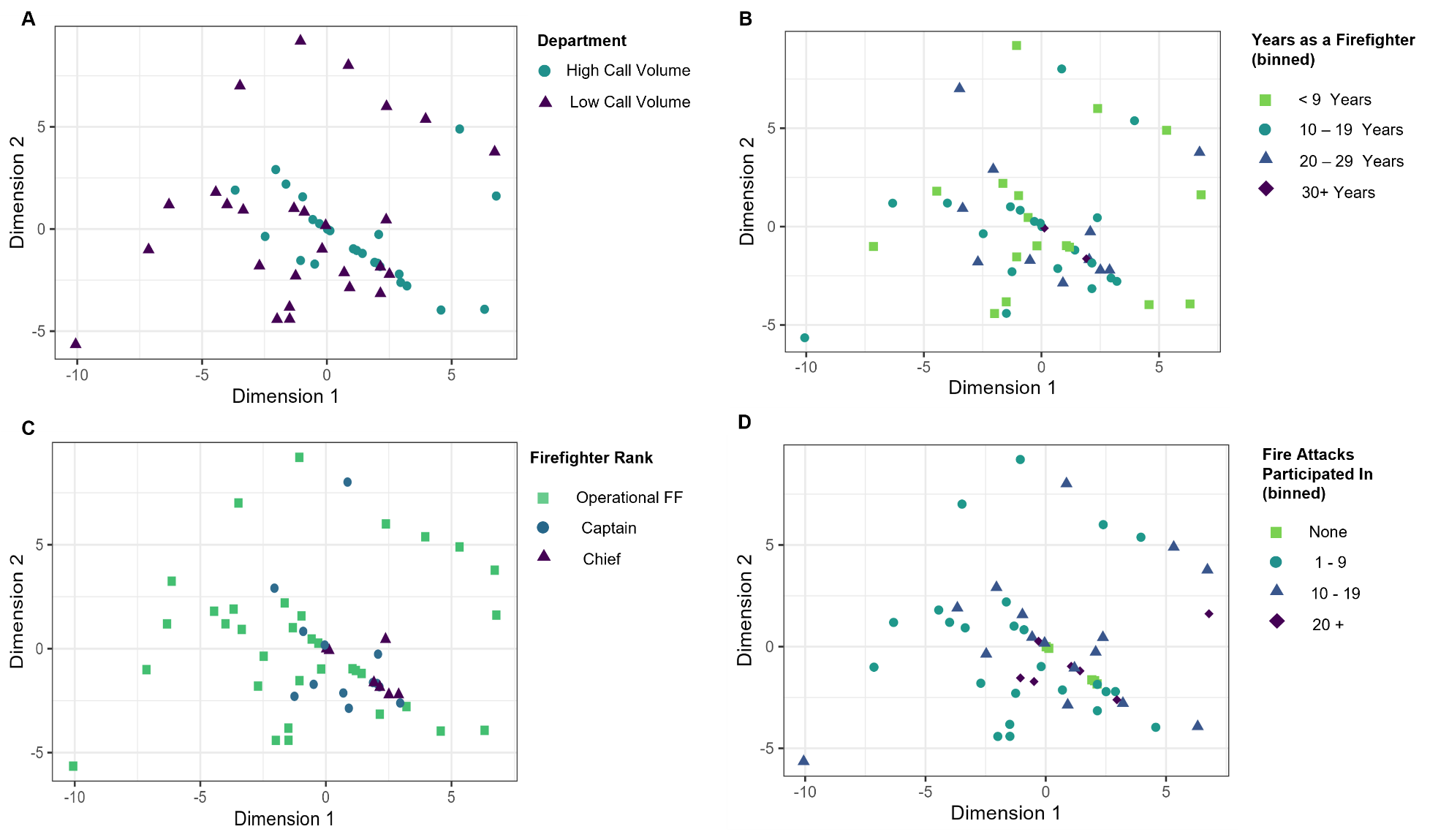** |
| --- |
| **Fig. S5** NMDS plots visualize sample similarity for occupationally relevant exposures (five FRs and one VOC), stress = 0.16. Exposure surrogates of **A** fire department, **B** years as a firefighter (binned into four categories: 0-9, 10-19, 20-29, 30+), **C** firefighter rank: chief, captain, and operational firefighter, and **D** fire attacks participated in (binned into four categories: 0, 1-9, 10-19, and 20+) are annotated with unique color and shapes. |


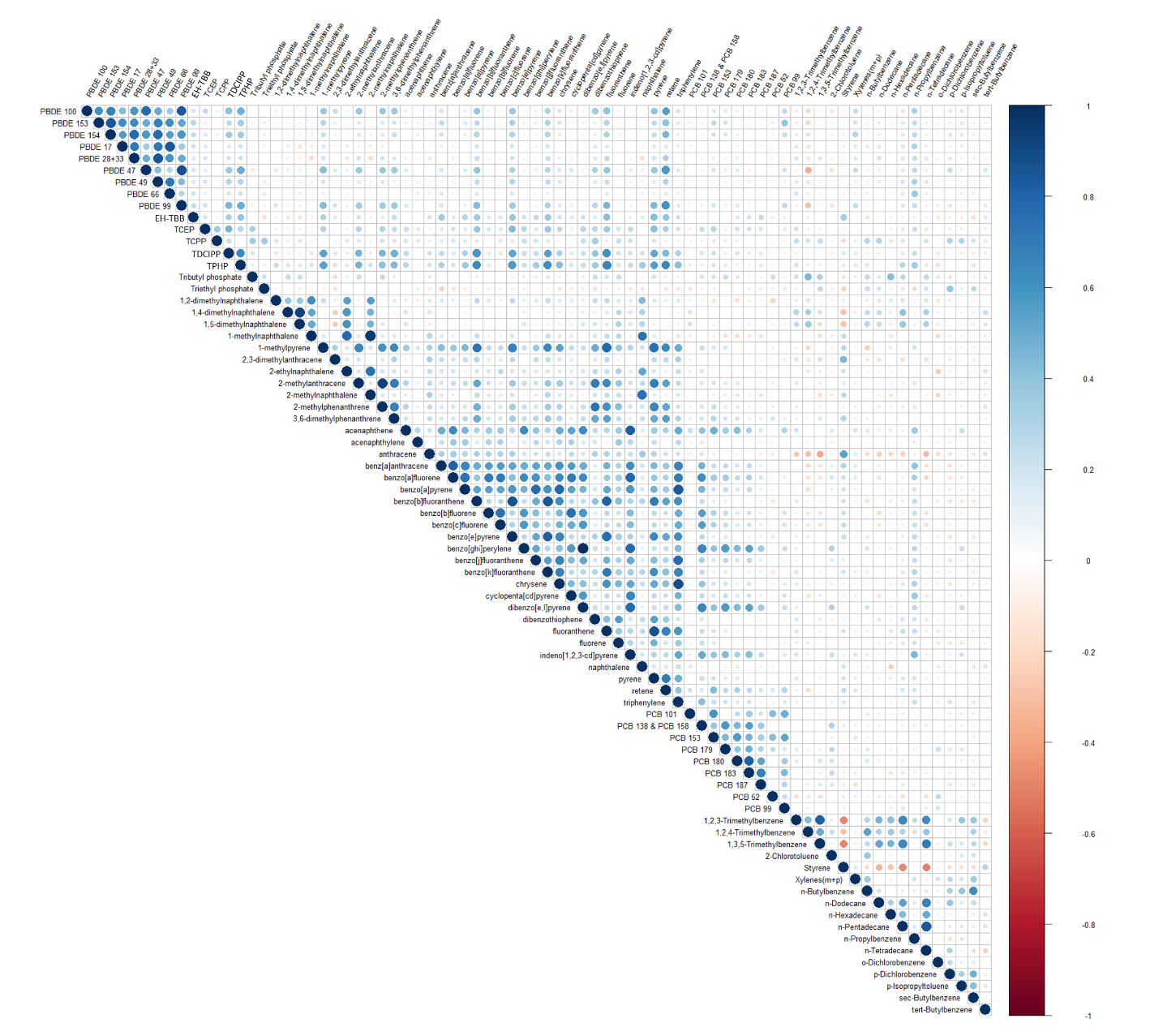


**Fig. S6** Spearman correlation matrix of ordinal exposure (very low, low, medium, or high) for all detected analytes.

# **References**

1. O’Connell SG, Kincl LD, Anderson KA. Silicone wristbands as personal passive samplers. Environmental science & technology. 2014;48(6):3327-35.

2. Kile ML, Scott RP, O’Connell SG, Lipscomb S, MacDonald M, McClelland M, et al. Using silicone wristbands to evaluate preschool children's exposure to flame retardants. Environmental research. 2016;147:365-72.

3. O’Connell SG, Anderson KA, Epstein MI. Determining chemical air equivalency using silicone personal monitors. Journal of exposure science & environmental epidemiology. 2022;32(2):268-79.
